# Seaweed for climate mitigation, wastewater treatment, bioenergy, bioplastic, biochar, food, pharmaceuticals, and cosmetics: a review

**DOI:** 10.1007/s10311-022-01520-y

**Published:** 2022-10-08

**Authors:** Mohamed Farghali, Israa M. A. Mohamed, Ahmed I. Osman, David W. Rooney

**Affiliations:** 1 Graduate School of Animal and Food Hygiene, Obihiro University of Agriculture and Veterinary Medicine, Obihiro, Hokkaido 080-8555 Japan; 2 Department of Animal and Poultry Hygiene and Environmental Sanitation, Faculty of Veterinary Medicine, Assiut University, Assiut, 71526 Egypt; 3 Graduate School of Animal and Veterinary Sciences and Agriculture, Obihiro University of Agriculture and Veterinary Medicine, 2-11 Inada, Obihiro, Hokkaido 080-8555 Japan; 4 School of Chemistry and Chemical Engineering, David Keir Building, Queen’s University Belfast, Stranmillis Road, Belfast, Northern Ireland BT9 5AG UK

**Keywords:** Seaweeds, Seaweed biogas, Seaweed biochar, Seaweed food, Climate change mitigations, Seaweeds cosmetics, Seaweeds pharmaceuticals, Biorefineries

## Abstract

The development and recycling of biomass production can partly solve issues of energy, climate change, population growth, food and feed shortages, and environmental pollution. For instance, the use of seaweeds as feedstocks can reduce our reliance on fossil fuel resources, ensure the synthesis of cost-effective and eco-friendly products and biofuels, and develop sustainable biorefinery processes. Nonetheless, seaweeds use in several biorefineries is still in the infancy stage compared to terrestrial plants-based lignocellulosic biomass. Therefore, here we review seaweed biorefineries with focus on seaweed production, economical benefits, and seaweed use as feedstock for anaerobic digestion, biochar, bioplastics, crop health, food, livestock feed, pharmaceuticals and cosmetics. Globally, seaweeds could sequester between 61 and 268 megatonnes of carbon per year, with an average of 173 megatonnes. Nearly 90% of carbon is sequestered by exporting biomass to deep water, while the remaining 10% is buried in coastal sediments. 500 gigatonnes of seaweeds could replace nearly 40% of the current soy protein production. Seaweeds contain valuable bioactive molecules that could be applied as antimicrobial, antioxidant, antiviral, antifungal, anticancer, contraceptive, anti-inflammatory, anti-coagulants, and in other cosmetics and skincare products.

## Introduction

Our planet faces several challenges, including climate change, rapid population growth, food shortages, and rising demand for bioactive compounds derived from nature in various aspects of life (Chen et al. 2022). To sustain these issues while simultaneously reducing negative effects on the ecosystem and preserving natural bioresources, deploying renewable biomass as a substitute for fossil fuels requires immediate and widespread adoption policies (Osman et al. 2021a). This may also involve the use of alternative renewable green energy sources.

**Fig. 1:**
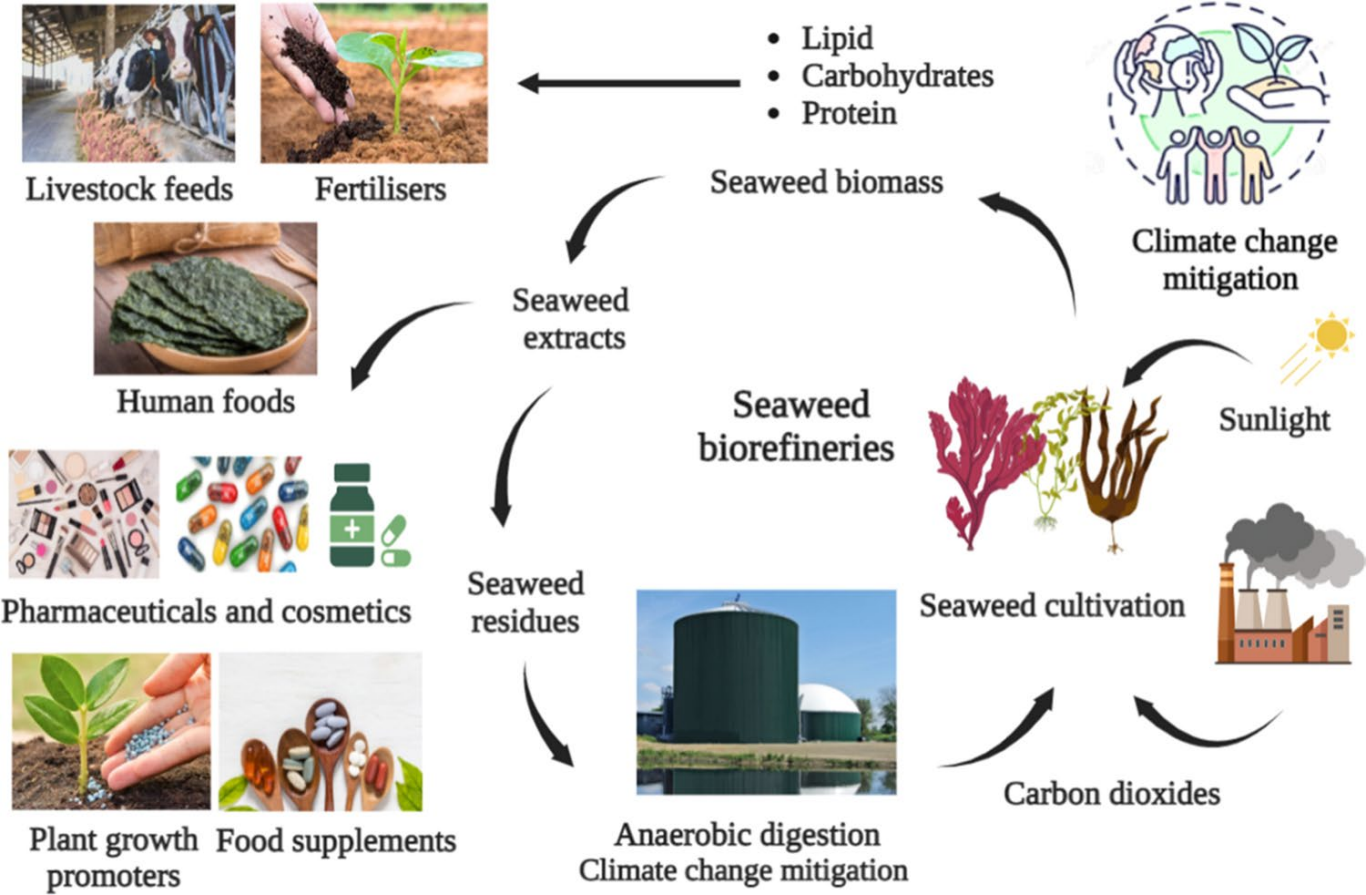
Seaweed biorefineries. Seaweeds can be harvested either through cultivation or from a natural source. Cultivated seaweeds use carbon dioxides from other refinery sources and the sun to sequester carbon within their biomass and are therefore regarded as a carbon sequestration tool when converted into a stable form of carbon such as biochar. In addition, wild seaweeds can float and descend deeper into the ocean, where they can be buried and act as a carbon sink. On the other hand, seaweeds can be extracted to obtain bioactive molecules that can be used in various biorefineries, such as antimicrobials, antioxidants, food supplements, plant growth promoters, anti-inflammatory, anticancer, contraceptives, cosmetics, and skin care agents. As a climate change mitigation strategy, seaweed residues or biomass can be used as feedstocks for anaerobic digestion to produce biomethane, which can be used to replace fossil fuels as a bioenergy source. Bioplastic derived from seaweed is an innovative method to replace synthetic, non-biodegradable plastics and protect the environment. Conversion of seaweed biomass to biochar is another method for mitigating climate change

Biomass biorefining for the production of diverse products, such as human food, animal feed, biochemicals, and bioenergy, through eco-innovative and sustainable bioprocess systems, is associated with sustainable development goals (Heimann 2019). Due to the biogenic origin of biomass, carbon dioxide emissions from bioprocesses do not contribute to a rise in atmospheric carbon dioxide levels (Tursi 2019; Osman et al. 2021b). Seaweeds are a rich source of unutilised biomass that can be used to address global challenges when cultivated using sustainable methods. As depicted in Fig. 1, seaweeds can address problems associated with climate change, bioenergy generation, agriculture, food consumption, animal and human health, useful chemicals, bioactive ingredients, and coastal management. In addition, if properly implemented, seaweeds could provide a sustainable circular bioeconomy strategy (Barbier et al. 2020).

Compared to lignocellulosic biomass from terrestrial plants, seaweeds are more suitable for biorefinery applications due to their rapid growth rates, extremely large yields, and lack of planetary land required for cultivation (Rajak et al. 2020). In addition, the absence of recalcitrant lignocellulosic assembles suggests that less energy may be required to recover high-valued bio-products of commercial interest, which favours economic and life cycle analyses of any assumed biorefinery bioprocess that uses seaweed as feedstocks. In addition, the existence of unique inherited polysaccharides in various seaweed species presents unique characteristics for either direct application or as compounds for the bioeconomy. Thus, seaweeds are third- or even fourth-generation feedstocks (Gaurav et al. 2017; Del Rio et al. 2020). However, the potential applications of seaweeds in biorefineries are still in their infancy, with progress beyond the laboratory scale being slow. Figure 1 depicts the review's interest in utilising seaweed biomass in various novel biorefineries. Specifically, the use of seaweeds in climate change mitigation and environmental sustainability, food consumption, animal feed additives, fish diets, bioplastic production, biofertilisers, biochar production, carbon sequestration tools, crop enhancers, antimicrobials, anti-inflammatory, anticancer, contraceptive, cosmetics, and skin care agents were reviewed.

The integration of seaweeds and bioprocesses can undoubtedly result in the commercialisation of seaweed biorefineries and call attention to the significant need for cooperative funding in this extremely promising research area, as well as the need for ongoing seaweed projects around the globe. In addition, the challenges currently faced by seaweed biorefineries and the future research required for seaweed's industrial growth are addressed.

## Worldwide seaweed production and seaweed types

### Global seaweed productions

Seaweeds are marine photosynthetic organisms, also known as “macroalgae,” that provide the energy foundation for all aquatic organisms, thereby playing a crucial role in the aquatic ecosystem's equilibrium. Multiple environmental benefits are provided by seaweed, such as carbon sequestration or capture, eutrophication mitigation, ocean acidification modification, shoreline protection, and habitat provision.

Seaweeds are an essential component of global aquaculture. In 2019, seaweed cultivation accounted for approximately 30% (wet weight) of the 120 gigatonnes of global aquaculture production, with brown (*Phaeophyceae*) and red (*Rhodophyta*) seaweeds, respectively, being the third- and second-largest contributors to global aquaculture after barbels, carps, and other cyprinids (FAO 2021a). Asia produces more than 97% of the world’s seaweed, with eight genera accounting for 96.80% of global seaweed production (Chopin and Tacon 2021).

In 1969, the 2.2 gigatonnes of global seaweed production were contributed equally by wild collection and cultivation, according to statistics. In 2019, cultivation seaweed production accounted for more than 97% of the world's seaweed production, while wild seaweed production remained at 1.1 gigatonnes (Cai et al. 2021). In Asia, more than 99.1% of seaweed production originated from cultivation, accounting for 97.38% of global production, with seven leading seaweed-producing nations in South or Eastern Asia, as shown in Table 1. Europe and the Americas accounted for 0.80 and 1.36%, respectively, of the world's seaweed production, with wild seaweed collection dominating, while cultivation accounted for only 3.87 and 4.70%, respectively, of total seaweed production. Contrarily, seaweed farming was the primary source of African seaweed production, accounting for 81.30 and 84.94% from Africa and Oceania, respectively; however, wild seaweeds only account for 0.41 and 0.05% of global seaweed production, respectively (Table 1).

**Table 1:** Global seaweed production

Area	Overall seaweed production (cultivated and wild)		Seaweed cultivation	
	Wet weight (thousand tonnes)	Global share (%)	Wet weight (thousand tonnes)	Share in cultivated and wild production (%)
*World*	35,762.5	100	34,679.1	96.97
*Asia*	34,826.8	97.383	34,513.2	99.10
China	20,296.6	56.754	20,122.1	99.14
Indonesia	9962.9	27.859	9918.4	99.55
Republic of Korea	1821.5	5.093	1812.8	99.52
Philippines	1500.3	4.195	1499.9	99.97
Japan	412.3	1.153	345.5	83.80
Korea	603	1.686	603	100.00
Malaysia	188.1	0.526	188.1	100.00
Rest of Asia	42	0.118	23.3	55.48
*Americas*	487.2	1.362	22.9	4.70
Chile	426.6	1.193	21.7	5.09
Peru	36.3	0.102	–	–
Canada	12.7	0.035	–	–
Mexico	7.3	0.021	0.01	0.14
USA	3.4	0.009	0.3	8.82
Rest of the Americas	0.904	0.003	0.9	99.56
*Europe*	287	0.803	11.1	3.87
Norway	163.2	0.456	0.1	0.06
France	51.5	0.144	0.2	0.39
Ireland	29.5	0.083	0.04	0.14
Russian Federation	19.5	0.055	10.6	54.36
Iceland	17.5	0.049	–	–
Rest of Europe	5.7	0.016	0.2	3.51
*Africa*	144.9	0.405	117.8	81.30
United Republic of Tanzania	106.1	0.297	106.1	100.00
Morocco	17.6	0.049	0.3	1.70
Madagascar	9.7	0.027	8.9	91.75
South Africa	11.2	0.031	2.2	19.64
Rest of Africa	.43	0.001	0.4	93.02
*Oceania*	16.6	0.046	14.1	84.94
Papua New Guinea	4.3	0.012	4.3	100.00
Solomon Islands	5.6	0.016	5.6	100.00
Kiribati	3.7	0.010	3.7	100.00
Australia	1.9	0.005	–	–
Rest of Oceania	1.1	0.003	0.6	54.55

There are 35.8 million metric tonnes of seaweed are produced worldwide. Around 97% of global seaweed production was originated from Asia. In Europe and the Americas, wild types of seaweed dominate production, whereas in Asia, Oceania, and Africa, cultivation dominates (Cai et al. 2021)

Even though seaweeds are generally low-value supplies, seaweed trades accounted for 5.4% of the $275 billion USA worth of world aquaculture production in 2019. This percentage was slightly lower than the other four groups, including cyprinids (carps and barbels), salmons, smelts, and trouts, marine prawns and shrimps, and crayfishes (Cai et al. 2021). The global market for commercial seaweed is anticipated to increase from $15.01 billion in 2021 to $24.92 billion in 2028. The global growth of the seaweed market can be attributed to the use of seaweed as a protective material against coronavirus, as highlighted by the World Health Organization, as well as the use of seaweeds in a variety of applications, including the food industry, livestock feed, agar, alginate, pharmaceutical, and others (Insights 2021).

In Eastern Asia, seaweeds are commonly consumed as human foods; however, in other world regions, seaweeds are consumed only by coastal communities or by very small numbers of consumers for a variety of purposes, such as exotic dietary foods, nutritional supplements (micronutrients), food with a low environmental footprint, and animal feed. In contrast, seaweed is not well known in several world regions.

In biorefineries, seaweeds have many applications, including foods and food supplements, animal feed, cosmetics, nutraceuticals, pharmaceuticals, textiles, biofertilisers/plant enhancers, biofuel, and bioplastic packaging, among others (FAO 2018). However, the contributions of seaweed to these products are typically dependent on the scientific community and seaweed-associated industries. Due to numerous environmental, social, and economic benefits and share, seaweed has the potential to contribute to various sustainable development goals (SDGs) such as sustainable development goals 1–3, 8, 10, and goals 12–14 (Duarte et al. 2021). Today, there is a growing interest in seaweed production, focussing on seaweed as a food resource to feed a growing human population and as a source of eco-friendly biomass (Cai et al. 2021).

### Seaweed types

In general, seaweeds are divided into three categories: brown seaweeds with over 2000 *Phaeophyceae* species, red seaweeds with over 7200 *Rhodophyta* species, and green seaweeds with over 1800 *Chlorophyta* species (Cai et al. 2021).

#### Brown seaweeds

The global cultivation of brown seaweed increased from 13 megatonnes in 1950 to 16.4 gigatonnes in 2019 at an average annual growth rate of 10.9%, which was higher than the global aquaculture growth rate (7.9%) for all species (Cai et al. 2021). In terms of tonnage and value, brown seaweeds accounted for 47.30 and 52.0%, respectively, of global seaweed cultivation in 2019, with Asia being the largest producer (99.93%). Kelp (*Laminaria/Saccharina*) and wakame (*Undaria*) are the most prevalent two genera of brown, cold-water seaweed worldwide. As shown in Table 2, seven nations supplied nearly 12.27 million tonnes of *Laminaria/Saccharina* in 2019, with 99.74% coming from four Asian nations and 0.27% coming from three European nations.

**Table 2:** Cultivation of various types of seaweed in 2019 (Cai et al. 2021; FAO 2021b)

Seaweed category	Seaweed family	Country/area	Production	
			Tonnes (wet weight)	Share of the world (%)
Brown seaweeds	*Laminaria*/*Saccharina*	*Worldwide*	12,273,748	100.00
		China	10,978,362	89.45
		Republic of Korea	662,557	5.40
		Korea	600,000	4.89
		Japan	32,600	0.27
		Faroe Islands	156	0.00
		Norway	73	0.00
		Spain	0.14	0.00
	*Undaria*	***Worldwide***	2,563,582	100.00
		China	2,023,930	78.95
		Republic of Korea	494,947	19.31
		Japan	44,600	1.74
		France	105	0.00
	Unidentified brown seaweeds	***Worldwide***	1,250,000	100.00
		China	1,240,000	99.20
		the Russian Federation	11,000	0.88
		USA	241	0.02
		Mexico	10	0.00
	*Sargassum* (mainly *S. fusiforme*)	***Worldwide***	304,000	24.32
		China	270,000	21.60
		Korea	34,000	2.72
	*Alaria esculenta*	***Worldwide***	105	0.01
		Norway	44	0.00
		Ireland	42	0.00
		Faroe Islands	19	0.00
	*Cladosiphon okamuranus*	Japan	90	0.01
	*Macrocystis pyrifera*	Chile	2	0.00
Red seaweeds	*Kappaphycus*/*Eucheuma*	***Worldwide***	11,622,213	100.00
		*Asia*	11,491,956	98.88
		Indonesia	9,795,400	84.28
		Philippines	1,498,788	12.90
		Malaysia	188,110	1.62
		China	4200	0.04
		Cambodia	2000	0.02
		Viet Nam	1700	0.01
		Timor-Leste	1500	0.01
		Sri Lanka	247	0.00
		Myanmar	11	0.00
		Africa	115,334	0.99
		United Republic of Tanzania	10,069	0.09
		Zanzibar	104,620	0.90
		Tanzania (mainland)	1449	0.01
		Madagascar	8865	0.08
		Kenya	400	0.00
		Oceania	14,050	0.12
		Solomon Islands	5600	0.05
		Papua New Guinea	4300	0.04
		Kiribati	3650	0.03
		Fiji	500	0.00
		Latin America and the Caribbean	874	0.01
		Brazil	700	0.01
		Saint Lucia	103	0.00
		Ecuador	45	0.00
		Grenada	20	0.00
		Belize	3	0.00
		Venezuela	3	0.00
	*Gracilaria*	***Worldwide***	3,639,833	100.00
		*Asia*	3,617,828	99.40
		China	3,480,850	95.63
		Indonesia	123,000	3.38
		Viet Nam	11,150	0.31
		Republic of Korea	1769	0.05
		Taiwan	976	0.03
		Philippines	83	0.00
		Latin America and the Caribbean	21,702	0.60
		Chile	21,672	0.60
		Brazil	30	0.00
		Africa	303	0.01
		Morocco	273	0.01
		Tunisia	30	0.00
		Europe	0.13	0.00
		Spain	0.13	0.00
	*Porphyra*	***Worldwide***	2,984,123	100.00
		*Asia*	2,984,123	100.00
		China	2,123,040	71.14
		Republic of Korea	606,873	20.34
		Japan	251,200	8.42
		Korea	3000	0.10
		Taiwan	10	0.00
Green seaweeds		Total 6 regions	16,696	100.00
	*Caulerpa* spp.	Philippines	1090	6.53
	*Monostroma nitidum*	Korea	6321	37.86
	*Capsosiphon fulvescens*	Korea	3386	20.28
	*Ulva* spp.	South Africa	2155	12.91
	*Codium fragile*	Republic of Korea	3258	19.51
	Green seaweeds nei	92.62% Viet Nam, 7.2% Portugal, and 0.18% Spain	486	2.91

With 18.25 gigatonnes, red seaweeds are the most abundant type of seaweed produced globally, followed by brown seaweeds with 16.40 gigatonnes and green seaweeds with 16.70 megatonnes. *Gracilaria*, *Eucheuma/Kappaphycus*, *Porphyra* are the most common red seaweed genera, while *Laminaria/Saccharina* and *Undaria* are the most commonly brown seaweed genera globally. Asia is the leading global brown and red seaweed producer, accounting for 99.93 and 99.17%, respectively

The majority of the 2.56 gigatonnes of *Undaria* (primarily *Undaria pinnatifida*) farming (7.40% of total seaweeds) were supplied by three Eastern Asian countries and one European country (0.004%) (Table 2). Farmed brown seaweed is mostly used for human consumption (e.g., wakame salads and kombu soup) as well as abalone feeds. Additionally, cultivated brown seaweed is used as a feedstock to produce (i) animal feeds; (ii) hydrocolloid (e.g., alginate for several biorefineries); (iii) biofertiliser or bio-stimulants; (iv) cosmetic or pharmaceutical ingredients; and (v) biodegradable bioplastics (FAO 2018).

#### Red seaweeds

The cultivation of red seaweeds increased from 21 megatonnes in 1950 to 18.25 gigatonnes in 2019, a 10.3% average annual increase that is less than brown seaweed but greater than global aquaculture growth (7.9%). In 2019, red seaweeds accounted for 52.65% of global seaweed cultivation, of which 99.17% occurred in Asia. Red seaweed cultivation is primarily dependent on two warm-water genera (*Gracilaria and Eucheuma/Kappaphycus*) and one cold-water genus (*Porphyra*, commonly called nori) (Cai et al. 2021). The 11.62 gigatonnes of *Eucheuma/Kappaphycus* cultivation in 2019, accounting for 33.54% of total seaweeds, was supplied by 23 regions, including nine Asian countries (98.88%), four in East African countries, four Pacific Islands, and six Latin American territories and the Caribbean, as shown in Table 2.

In 2019, 99.40% of the 3.64 gigatonnes of cultivated *Gracilaria* (10.50% of total seaweed production) were produced by Eastern and South-eastern Asia (Table 2). The 2.98 gigatonnes cultivated *Porphyra* represented 8.61% of seaweed produced by five Eastern-Asian countries (Table 2). *Gracilaria* are typically used for agar generation and abalone feeds, whereas *Eucheuma*/*Kappaphycus* are mainly employed to isolate carrageenan (FAO 2018). In addition to alginate purified from brown seaweeds, carrageenan and agar are hydrocolloids derived from seaweed that are commonly used in food and/or non-food biorefineries. *Eucheuma*/*Kappaphycus* and *Gracilaria* are also used as human foods (such as pickles and salads), while *Porphyra* are primarily used in sushi wrap and as a soup ingredient.

#### Green seaweeds

Since 1990, green seaweed cultivation has been comparatively smaller and on a downward trend. The 16.70 megatonnes of global green seaweed farming in 2019 represented approximately 0.048% of total seaweed production, which was less than half of the maximum production in 1992 (38.6 megatonnes). This is in contrast to the rapid growth of brown seaweed cultivation (3-folds) and red seaweed cultivation (15-folds) over the same time period (Cai et al. 2021).

In 2019, six seaweed species cultivated an average of more than 500 kilograms of green seaweed. During 1950–2019, *Caulerpa spp.* was the most abundant green seaweed species, with an average annual production of 6.4 megatonnes; however, the Philippines’ contribution decreased from 28.7 megatonnes in 1998 to 1.09 megatonnes in 2019. In 2019, the total production of *Monostroma nitidum* was 6.3 megatonnes, which was lower than the maximum production of 17.7 megatonnes in 1992 (Cai et al. 2021).

In 2019, the Republic of Korea cultivated *Capsosiphon fulvescens*, *Monostroma nitidum*, and *Codium fragile*, which accounted for 12.97 megatonnes of the global green seaweed harvest and 78% of the global green seaweed total (see Table 2). Green seaweeds that have been cultivated can be used as vegetables in salads. Both *Caulerpa lentillifera* (green caviar or sea grape) and *Monostroma nitidum* (green laver) are considered delicacies in the marketplace. Other uses for green seaweeds include biofertiliser, animal feeds, bio-stimulants, cosmetics, pharmaceuticals, and wastewater treatment (FAO 2018).

### Wild seaweeds harvest

From approximately 1.06 gigatonnes in 2006 to a maximum of 1.29 gigatonnes in 2013 and settling at approximately 1.09 gigatonnes (wet weight) in 2015, wild harvests have remained constant (FAO 2018). In 2015, Chile produced the most wild seaweed (345.704 megatonnes), followed by China (261.77 megatonnes), Norway (147.39 megatonnes), and Japan (93.3 megatonnes).

The dominant species harvested from the wild seaweeds are Chilean kelp (*Lessonia nigrescens*) with 22% of the total harvested species, followed by huiro palo (*Lessonia trabeculata*) with 7%, *Gracilaria* spp. with 5% and the rest-tangle (*Laminaria digitata*), luga negra (*Sarcothalia crispata*), kelp (*Macrocystis* spp.), Japanese kelp (*Saccharina japonica*), North Atlantic rockweed (*Ascophyllum nodosum*), and *Gigartina skottsbergii*—accounting for less than 5%. Farmed and wild *Gracilaria* species are a major source of agar for human consumption (FAO 2018).

Contamination by heavy metals such as mercury and arsenic is a significant concern with wild seaweed. These factors inhibit market expansion, particularly in nations prioritising food safety and sustainability. Consumers will be willing to pay more for seaweed from nations with a strict coastal zone management policy (FAO 2018).

### Chemical components of seaweed

The classification of seaweeds into three major taxonomic groups was made possible by morphological and pigment characteristics (red, brown, and green seaweed). Seasonally and geographically variable carbohydrates, lipids, proteins, minerals, and vitamins are present in seaweeds (Torres et al. 2019). Due to their complex composition, their hydrocolloids or polysaccharides, such as agars, alginates, and carrageenan, seaweeds can also be utilised in various biorefineries.

Seaweeds contain 70–90% water (fresh weight basis) and are primarily composed of 25–77% carbohydrates (dry matter basis), 5–43% proteins (dry matter basis), 9–50% ash content (dry matter basis), and 1–5% lipids (dry matter basis) (Del Rio et al. 2020; Praveen et al. 2019). The major carbohydrates presented in seaweed are cellulose, sucrose, starch, carrageenan, ulvan, laminarin, mannitol, agar, fucoidan, and alginate (Del Rio et al. 2020). The absence or low lignin content of seaweed, as low as 0.03 g/kg dry weight (Wang et al. 2020a; Ghadiryanfar et al. 2016), facilitates biofuel processing and degradation compared to the costly pretreatment required for traditional lignocelluloses biomass (Elsayed et al. 2019). In addition, the high carbohydrate and low lipid content of seaweeds make them ideal candidates for alcohol-based biofuels (Sirajunnisa and Surendhiran 2016). Table 3 shows the primary components of various seaweeds.

**Table 3:** Chemical components of seaweeds

Group	Seaweed species	Protein (dry weight%)	Carbohydrate (dry weight%)	Lipid (dry weight%)	Ash content (%)	References
Brown seaweed	*Ascophyllum nodosum*	4.8–9.8	39.5–60.6	1.9–4.8	18–24	Ghadiryanfar et al. (2016)
	*Fucus serratus*	9.6	26.4	2.8	18.8	Kostas et al. (2016)
	*Laminaria digitata*	26.8	21.7	1.9	24.3	Kostas et al. (2016)
	*Dictyopteris australis*	9.70	33.12	1.34	28.11	Verma et al. (2017)
	*Stoechospermum marginatum*	10.90	33.58	3.91	35.83	Verma et al. (2017)
	*Iyengaria stellata*	11.73	31.96	2.84	31.17	Verma et al. (2017)
	*Sargassum linearifolium*	8.93	29.82	1.93	31.5	Verma et al. (2017)
	*Laminaria digitata*	12.9	46.6	1.0	26.0	Kostas et al. (2017)
	*Saccharina japonica*	8	51	1	Not mentioned	Jambo et al. (2016)
	*Undaria pinnatifida*	24	43	4	Not mentioned	Jambo et al. (2016)
	*Stypopodium schimperi*	1.12–3.15	Not mentioned	2.48–11.53	3.88–17.98	Lee et al. (2020)
	*Sargassum thunbergii GEEL-15*	7.14	37.0	7.88	20.84	Yang et al. (2021a)
	*Sargassum vulgare*	10.32	39.07	4.02	30.09	de Melo et al. (2021)
Red seaweed	*Palmaria. palmata*	22.9	39.4	3.3	25.7	Kostas et al. (2016)
	*Ulva lactuca*	16.4	23.8	1.0	21.5	Kostas et al. (2016)
	*Dictyota dichotoma*	9.52	35.11	2.67	40.13	Dixit et al. (2018)
	*Sciania fasciularis*	8.07	22.99	0.97	23.232	Verma et al. (2017)
	*Gelidium micropertum*	9.13	37.81	2.20	15.678	Verma et al. (2017)
	*Halymenia venusta*	14.13	34.81	1.43	17.123	Verma et al. (2017)
	*Rhodymenia dissecta*	9.84	33.87	1.45	21.163	Verma et al. (2017)
	*Haloplegma duperreyi*	9.33	30.50	0.55	17.12	Lee et al. (2020)
	*Halymenia venusta*	14.13	34.81	1.43	17.12	Lee et al. (2020)
	*Gracilaria gracilis*	13.70	28.6	1.70	36.00	Lee et al. (2020)
	*M. stellatus GEEL-16*	9.14	35.08	4.63	28.17	Yang et al. (2021a)
	*Gracilaria corneus*	21.27	23.55	1.93	34.16	de Melo et al. (2021)
	Hypnea valentiae	4.56	30.10	0.73	32.88	Dixit et al. (2018)
	*Acanthophora spicifera*	6.55	48.51	1.40	47.04	Dixit et al. (2018)
	*Gracilaria corticata*	5.46	33.29	0.88	28.51	Dixit et al. (2018)
	*Corallina mediterranea*	10.7–20.6 (17.05)	24.4–29.4 (26.6)	1.49–2.55 (2.07)	35.5–42.1 (39.0)	Mohy El-Din (2018a)
	*Gracilaria gracilis*	13.7	28.6	1.7	36	Parsa et al. (2018)
Green seaweed	*Ulva lactuca*	12.17	32.61	1.45	20.940	Verma et al. (2017)
	*Acrosiphonia orientalis*	7.47	24.55	1.24	24.980	Verma et al. (2017)
	*Valonia utricularis*	9.03	12.80	3.69	20.317	Verma et al. (2017)
	*Ulva* sp. GEEL-17	4.24	55.40	6.67	21.30	Yang et al. (2021a)
	*Ulva fasciata*	11.42	40.91	2.37	20.89	de Melo et al. (2021)
	*Ulva fasciata*	6.55	44.51	2.45	24.92	Dixit et al. (2018)
	*Caulerpa sertularioides*	9.44	44.7	1.88	31.24	Dixit et al. (2018)
	*Ulva lactuca*	15.65–23.2 (Average 19.34)	17.2–19.5 (18.50)	2.28–4.05 (3.46)	21.5–28.33 (25.8)	Mohy El-Din (2018a)
	*Cladophora glomerata*	13.70	34.70	2.40	26.10	Parsa et al. (2018)
	*Caulerpa macrodisca*	20.54	37.66	1.42	29.03	Zuldin et al. (2021)

Seaweeds pose unique structural properties in terms of carbohydrates, protein, and lipids. Seaweeds contain 12.8–60.6% carbohydrates (dry matter basis), 1.12–26.8% proteins (dry matter basis), 3.88–40.13% ash content (dry matter basis), and 0.55–11.53% lipids (dry matter basis). Variations in chemical components of seaweeds are attributed to different species, collection seasons, and growing environments

Brown seaweeds (*Phaeophyceae*) are olive-greenish to dark brownish due to the presence of fucoxanthin pigments, which mask the original chlorophyll colour. The brown seaweeds include kelp (*Laminaria spp.*), which can attain a maximum length of 100 m and a daily growth rate of 50 cm (Sudhakar et al. 2018; Wei et al. 2013). 55% (dry weight basis) of brown seaweeds are composed of laminin and mannitol as storing polysaccharides (Hreggviðsson et al. 2020). Laminarin is a polysaccharide that may be hydrolysed into glucose sugar monomer by laminarase (endo-1,3(4)-b-glucanase) (Del Rio et al. 2020). Mannitol can dehydrogenate into fructose, which can be further bio-converted into bioethanol (Wang et al. 2020a; Horn et al. 2000). In addition, brown seaweeds contain alginate and cellulose, which are fundamental polysaccharides that give the cell wall mechanical strength. Typically, high levels of total carbohydrates (up to 65%) make brown seaweeds attractive biomass for biofuel purposes (Del Rio et al. 2020).

Due to the presence of phycoerythrin and phycocyanin pigments, red seaweed (*Rhodophyceae*) has a characteristic red/pink colour. These seaweeds can grow in depths ranging from 40 to 250 m (Wang et al. 2020a). 40–70% (dry weight basis) of red seaweeds are composed of carbohydrates, such as glucan, cellulose, and galactan (Praveen et al. 2019). The structural cell wall of red seaweeds contains carrageenan and agar, which are valuable long-chain polysaccharides for gel formation and thickening foods such as ice cream, yoghurt, and pudding (Samaraweera et al. 2012; Zhang et al. 2019a).

Green seaweeds (*Chlorophyceae)* typically grow as paper-thin sheets or filamentous springy fingers in shallow, near-surface water. Green seaweeds contain photosynthetic pigments, such as carotenoids and chlorophyll A and B. *Chlorophyceae* mainly consist of 40 and 60% dry matter polysaccharides, including starch, pectin, and cellulose (Praveen et al. 2019; Michalak 2018). Because of variations in environmental conditions, the chemical composition of seaweeds varies considerably between species and seasons. For instance, *Ulva* sp. contained the maximum carbohydrates contents in June (61% dry weight basis), while the same species exhibited a steady decline from 49 to 41 dry weight% throughout July to September, respectively (Wang et al. 2020a). Similarly, *Ulva intestinalis* presented a peak protein content of 27.7% in the winter, which dropped to 6.7% in the spring (Osman et al. 2020).

Furthermore, the extensive seasonal variation in water properties results in substantial variations in seaweed biomass yields. For instance, *Ulva intestinalis* had the maximum biomass yield of 61.5 g/square metre/year, while *Ectocarpus siliculosus* showed 1.3 g/square metre/year (Osman et al. 2020). In order to determine the optimal yield period for seaweeds, the variety of seaweed must be determined based on the season, the growth cycle, and the desired end products.

### Economic benefits of the seaweed industry

The global cultivated seaweed production from the 34.7 gigatonnes for various biorefineries valued at 14.7 billion United States dollars, which mainly contributed to *Laminaria*/*Saccharina* (4.6 billion United States dollars), *Porphyra* (2.7 billion United States dollars), *Kappaphycus*/*Eucheuma* (2.4 billion United States dollars), *Gracilaria* (2 billion United States dollars) and *Undaria* (1.9 billion United States dollars). In 2019, average first-sale estimates were 0.47 United States dollars/kilogram (wet weight) for brown seaweeds, 0.39 United States dollars/kg for red seaweeds and 0.79 United States dollars/kg for green seaweeds (Cai et al. 2021).

Seaweed cultivation is usually a labour-intensive industry that employs a large number of people. Therefore, a substantial portion of a first-sale price’s $14.7 billion is converted into wages supporting various households' incomes in coastal areas. Additional downstream activities, such as postharvest handling, processing, distribution, and marketing, generate more jobs and income. Additionally, carrageenan extraction from seaweed created numerous administrative and support positions in government offices and laboratories (Cai et al. 2021).

According to United Nations Comtrade statistics, 98 nations earned 2.65 billion United States dollars of foreign exchange in 2019 through exporting seaweeds (909 million United States dollars) and seaweed-based hydrocolloids (1.74 billion United States dollars). For instance, China, Indonesia, the Republic of Korea, Philippines, Chile, Spain, France, the USA, Germany, and the UK have gained approximately 578, 329, 320, 252, 209, 145, 124, 102, 82, and 78 million United States dollars from exporting of seaweeds and seaweed-based hydrocolloids in 2019, respectively (Cai et al. 2021).

The protein content of seaweeds ranges from 10 to 30% (based on dry matter content), with red and green seaweeds typically containing more protein than brown seaweeds. The lipid content of seaweed ranges between 1 and 5% of seaweed’s dry matter. The levels of protein and lipids in seaweeds varied by harvest season. 500 gigatonnes of dry seaweed would yield 100 and 15 gigatonnes of seaweed protein and oil, respectively, assuming a lipid content of 3% and a protein content of 20% (Table 4). Comparable to soy protein when considering the amino acid content and anti-nutritional properties of both soy protein and seaweed. Taking into account the profile of long-chain omega-3 fatty acids makes seaweeds more advantageous than other soy proteins and comparable to the nutritional value of fish oils. Currently, about 250 gigatonnes of soy protein and 1 gigatonne of fish oil are produced annually. Consequently, 500 gigatonnes of seaweeds could replace nearly 40% of current soy protein production and represent a 750% increase over fish oil. Utilising seaweeds and seaweeds containing oils would provide long-chain omega-3 fatty acids that are beneficial to human health and could eliminate the need for fish oil in animal feeds and aquaculture.

**Table 4:** Generalised services from 500 gigatonnes dry weight of seaweeds (World-Bank-Group 2016)

Item	Service/production	Remarks
Ocean area required	500,000 km^2^ (About 0.03% of the ocean surface areas)	Estimated from average annual production of 1000 dry tonnes/square kilometre
Protein yield	100 gigatonnes	Based on the protein content of 20%/dry seaweed’s weight
Algal oil for people and animals	15 gigatonnes	Based on the lipid content of 3%/dry seaweed’s weight Provides 23 United States billion dollars that would completely substitute fish oil in animal diets
Nitrogen removal	1 gigatonne	Assumes nitrogen content of 0.2% of dry weight Represent 61% of the nitrogen input as fertiliser
Carbon removal	135 gigatonnes	Based on seaweed’s carbon content of 27%/dry weight. Equivalent to 6% of the carbon input annually to oceans
Bioenergy potential	1250 terawatt-hour	Based on 50% carbohydrate content, bio-converted to bioenergy. Equivalent 1% of annual world energy utilise
Land sparing	1,000,000 square kilometres	Presumes five tonnes/hectare farm yields. Equivalent 6% of world cropland
Freshwater saving	500 cubic kilometres	Presumes agricultural use about 1 cubic metre water/kilogram biomass. Equivalent 14% of annual world freshwater withdrawals

Seaweeds have the potential to supply 500 gigatonnes of dry seaweed that would generate 100 and 15 gigatonnes of seaweed protein and oil, respectively. In addition, these quantities can eliminate 1 gigatonne of nitrogen, 135 gigatonnes of carbons, 1250 terawatt-hours of energy, and 500 cubic kilometres of fresh water

The prices of soy meal and fish oil are approximately $550 and $1500 per tonne, equating to approximately $28 and $15 billion for the protein and oil fractions of seaweed. Approximately one job per 10 tonnes of dry seaweed can be generated; therefore, the seaweed industry must generate approximately 50 million jobs in addition to the 100 million jobs generated by marine capture fisheries (World-Bank-Group 2016).

### Summary

Nearly 34.65 gigatonnes, or approximately 30% of the 120 gigatonnes of global aquaculture production, come from seaweed cultivation. There are three types of seaweed: brown, red, and green. The first two types represent 16.40, and 18.25 gigatonnes, respectively. Asia produces approximately 97.4% of the world’s seaweed, of which 99.1% is cultivated. Europe and the Americas produced 0.80 and 1.36%, respectively, of the world's seaweed, with wild seaweed dominating.

In biorefineries, seaweeds have many applications, including foods and food supplements, animal feed, cosmetics, nutraceuticals, pharmaceuticals, textiles, biofertilisers/plant enhancers, biofuel, and bioplastic packaging, among others. Seaweed has the potential to contribute to several sustainable development goals, including goals 1–3, 8, 10, and 12–14. In general, seaweeds are comprised of 70–90% water, 25–77% carbohydrates (dry matter basis), 5–43% proteins (dry matter basis), 9–50% ash content (dry matter basis), and 1–5% lipids (dry matter basis). Seaweed contains cellulose, sucrose, starch, carrageenan, ulvan, laminarin, mannitol, agar, fucoidan, and alginate as seaweed’s primary carbohydrates. Compared to traditional lignocellulosic biomass, seaweed’s low lignin content makes biomass processing and degradation simpler from a biofuel standpoint.

## Environmental benefits of seaweed cultivation

### Role of seaweed in climate change mitigation

**Fig. 2:**
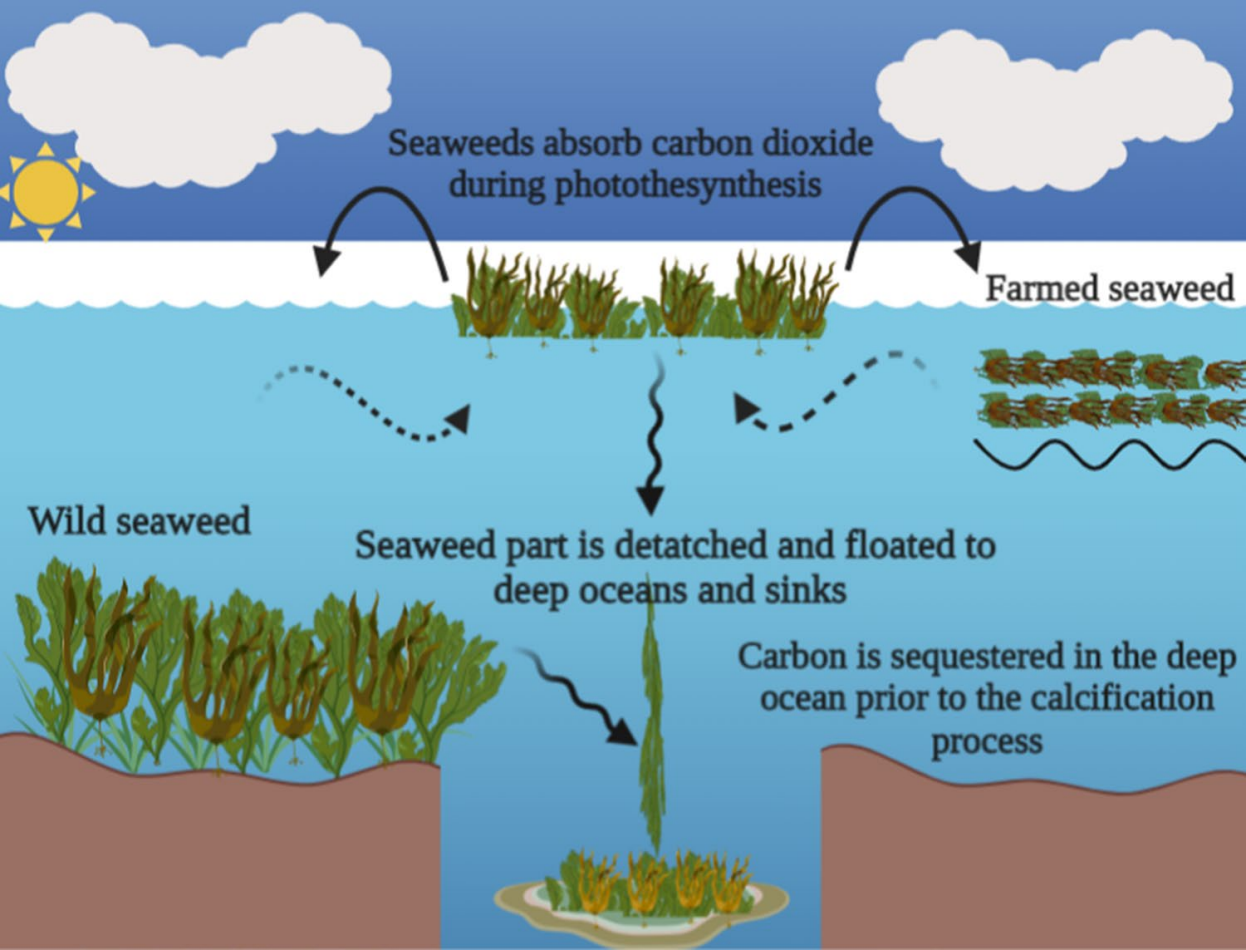
Seaweed’s role in deep ocean carbon sequestration, which is an effective carbon sequestration strategy. Seaweeds have the capacity to remove carbon dioxide from the atmosphere. Then, there are two modes for transporting seaweeds to the sediment and depths of the ocean: the drift of seaweed particles through marine canyons and the sinking of negatively floating seaweed detritus. Overall, seaweeds can store 173 teragrams of carbon per year on average

**Fig. 3:**
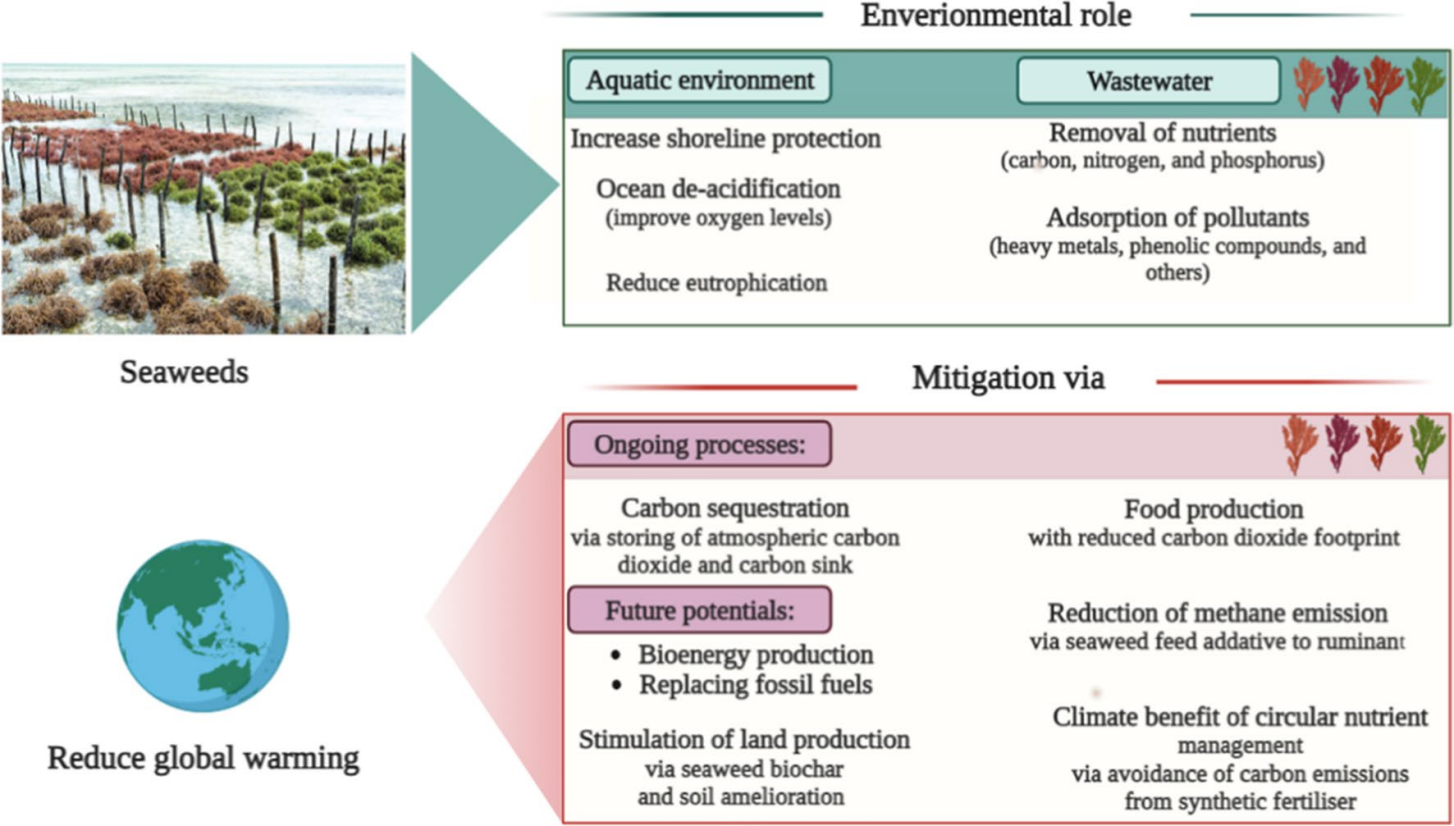
Beneficial functions of seaweeds in environmental restoration and climate change mitigation. Therefore, seaweeds can be viewed as carbon sequestration tools due to their ability to reduce carbon footprint. Seaweeds have the capacity to restore water pH, oxygen levels, and shoreline protection against wave energy dissipation. In addition, using seaweed biomass as feedstocks for biogas production is a promising area of research that can be utilised to replace fossil fuels. Utilising seaweeds for biochar production is also a promising area of research for the environmental sequestration of carbon and the benefit of plants

As a result of increased carbon dioxide emissions, global temperatures are increasing. Currently, the situation is deteriorating, particularly due to the rapid economic growth of developing nations whose carbon dioxide emissions are anticipated to rise in the near future. Therefore, taking all feasible measures to reduce atmospheric carbon dioxide load to prevent ecological damage is essential (Jhariya et al. 2021; Banerjee et al. 2021a, b). To replace fossil derivatives, climate change has prompted a blue carbon paradigm in which food and fuel can be obtained from aquatic environments through carbon harvesting, carbon sequestration, and carbon sinking (Yong et al. 2022). Seaweeds have the potential to serve as a renewable energy source and carbon sink; furthermore, seaweeds may play a significant role in climate change mitigation strategies, as shown in Figs. 2 and 3.

Blue carbon emphasises the capture and storage of organic carbon by the oceans and coastal environments, with coastal vegetated ecosystems contributing significantly to global carbon sequestration (Macreadie et al. 2019). Particularly, seaweed may absorb a significant amount of carbon dioxide from the aquatic ecosystem and support a variety of ecological benefits, such as remediation of shore contaminants and habitat for other aquatic organisms (Macreadie et al. 2019; Duarte et al. 2017a). Yong et al. (2022) recently reported on the potential contribution of seaweed to the newly emerging blue carbon strategy and seaweed's role in mitigating climate change over the long term. The authors reported that seaweed possessed all the necessary characteristics for classification as a blue carbon reservoir with a substantial carbon sink potential, in addition the role of seaweed in climate change mitigation, bio-economy enhancement via fossil fuel substitution, human food, biofuels, renewable biomass, and animal feed. About 50% of the world's carbon could be sequestered by seaweed (Chung et al. 2011; Jagtap and Meena 2022). In addition, seaweed can offset half of the world's bioenergy, making seaweed a potential means of reducing greenhouse gas emissions (Duarte et al. 2017b).

Numerous studies have highlighted seaweed’s capacity as a carbon sink (Yong et al. 2022; Macreadie et al. 2019; Moreira and Pires 2016; Krause-Jensen and Duarte 2016). Krause-Jensen and Duarte (2016) stated that seaweeds grown in coastal zones are effectively sequestered carbon dioxides from the atmosphere and act as a carbon sink organism in deep oceans and marine sediments. Globally, they estimated that seaweeds could sequester between 61 and 268 megatonnes of carbon per year, with an average of 173 megatonnes. Nearly 90% of carbon was sequestered by exporting biomass to deep water, while the remaining 10% was buried in coastal sediments. The 173 megatonnes of carbon per year sequestered by wild seaweeds are dispersed throughout the deep ocean, where the carbon supplying this flux is produced over 3.5 million square kilometres inhabited by seaweed (Krause-Jensen and Duarte 2016). Aquaculture of seaweed has the potential to sequester approximately 1500 tonnes of carbon dioxide per square kilometre, which is equivalent to the annual carbon dioxide emissions of approximately 300 Chinese individuals (Duarte et al. 2017a). Lehahn et al. (2016) demonstrated that the cultivation of seaweeds could completely replace the reliance on fossil fuels for transportation, meet 100% of the future demand for acetone, ethanol, and butanol, provide 5–24% of the demand for proteins and produce biogas that could mitigate 5.1 × 10^7^–5.6 × 10^10^ tonnes of carbon dioxide emissions from natural gas use.

Jagtap and Meena (2022) reported the carbon sequestration potential of certain seaweeds as follows: *Eucheuma* spp. can sequester 68.43 tonnes carbon/hectare/year, *Kappaphycus striatum* can sequester 125.51 tonnes carbon/hectare/year, *Laminaria* spp. can sequester 1156 tonnes carbon/hectare/year, *Ecklonia* spp. can sequester 562 tonnes carbon/hectare /year, *Sargassum* spp. can sequester 346 tonnes carbon/hectare/year, and *Gelidium* spp. can sequester 17 tonnes carbon/hectare/year. The authors reported that the overall carbon sequestration by seaweed cultivation in Indonesia was 621,377 tonnes of carbon/year and 2.66 million tonnes of carbon/year from the pond and marine culture, respectively. Thus, seaweed can sequester carbon and reduce atmospheric carbon dioxide levels, thereby mitigating the effects of global warming. In addition to carbon sequestration, seaweed acquires nutrients from water bodies, where seaweed uses nitrogen and phosphorus and fixes carbon in the water through photosynthesis, which has multiple benefits, including reducing carbon and nitrogen concentrations in the water, mitigating ocean acidification, and increasing oxygen levels to revitalise and restore water habitats (Yong et al. 2022).

The major limitations of this claim stem from the notion that a carbon sink concept should be provided by carbon buildup in seaweed's biomass; however, seaweed's carbon consumed as human food or fed to livestock enters the carbon cycle and provides no carbon sink meaning (Troell et al. 2022). Therefore, when seaweeds are transferred to the deep ocean and sediments, they are considered a carbon sink or converted into biochar. Optimising the blue carbon role of seaweeds necessitates the management of the seaweed’s fate, whether seaweed originated from aquaculture or was harvested in the wild, in order to address this issue. One option is to replace fossil fuels with biofuels produced from seaweed biomass (Chen et al. 2015; Farghali et al. 2021; Ap et al. 2021) or substitute food/feed production practices of intensive carbon dioxide footprints with seaweed-established food/feed means, which has much lower life-cycle carbon dioxide emissions (Duarte et al. 2017a; Troell et al. 2022). Another decarbonisation pathway uses seaweed to reduce enteric methane emissions from ruminants (Troell et al. 2022).

In addition to acting as a carbon sink, seaweed is an excellent candidate for removing carbon dioxides from the atmosphere due to seaweed’s rapid growth rate and high photosynthetic efficiency (Sondak et al. 2017). The carbon dioxides emitted from the carbon-based power plant's combustion may be injected into closed or open seaweed systems in order to increase seaweed growth rate and carbon sequestration (Cole et al. 2014). During cultivation, one tonne of dry seaweed biomass can absorb nearly 960 kilograms of carbon dioxide. Additionally, seaweed has additional eco-benefits, such as reducing global warming, eutrophication, and acidification. Seaweed can also be used to fixate phosphorus, potassium, and nitrogen (Duarte et al. 2017b).

### Seaweed as a potential wastewater treatment tool

Industrial effluents and aquaculture farms typically cause severe environmental issues, such as intense pollution and ecological degradation. The presence of significant nutrients in water bodies, such as nitrogen and phosphorus, frequently results in water eutrophication, which results in hypoxia and the prevalence of harmful microalgal blooms (Arumugam et al. 2018).

The most effective way to reduce pollution is to treat wastewater at the pollution’s source; however, most industries and aquacultures lack on-site treatment technologies (Wang et al. 2020a). In most cases, chemical, physical, and biological methods are used to treat wastewater (Tawfik et al. 2022a). Biological processes are superior to other treatment methods due to their straightforward operation, low cost, and eco-friendliness. Seaweeds can be used for the biological removal of phosphorus and nitrogen from wastewater (Fig. 3). Seaweeds can utilise ammonia–nitrogen and nitrate, two common nitrogen compounds found in agricultural, industrial, and sewage water discharges (Wang et al. 2020a). Xiao et al. (2017) estimated the role of large-scale seaweed farms in removing nutrients and mitigating coastal water eutrophication in China. They found that seaweed farming removed approximately 75 and 9.5 megatonnes of nitrogen and phosphorus, respectively, in China. The authors projected that the seaweed industry would eliminate 100% of the total phosphorus feed into Chinese coastline waters by 2026. The World Bank estimates that a global seaweed harvest of 500 million tonnes by 2050 will be achieved, which will utilise approximately 10 million tonnes of water nitrogen, which represents 30% of the nitrogen that reaches the seas, and 15 million tonnes of phosphorus, which is about 33% of the phosphorus generated from dung and fertilisers (Jagtap and Meena 2022). Duan et al. (2019) showed that *Gracilaria lemaneiformis* cultivation could sequester 1192.03 tonnes of carbon, 15.89 tonnes of phosphorus, and 128.10 tonnes of nitrogen from the Yantian Bay seawater.

Utilising fungi and bacteria for bioremediation has been intensively studied and is currently attracting significant interest. However, growing microorganisms require external carbon sources for optimal growth (Wang et al. 2020a). Due to their autotrophic growth, seaweeds are promising bioremediation agents. The cell walls of seaweed are composed of multiple polymers, including cellulose, pectin, hemicellulose, and arabino-galactan proteins. The predominant functional groups consisting of carboxyl, amines, and phosphoryl provide negative charges to the cell walls of the seaweed, thereby attracting pollutants with cationic groups to the seaweed's surface and initiating the sorption process (Wang et al. 2020a). Bioaccumulation was primarily responsible for the seaweed's uptake of organic contaminants and other growth supplements. Table 5 details the ability of seaweed to absorb certain heavy metals from bodies of water.

**Table 5:** Seaweed’s role in removing heavy metals, phosphorus, and nitrogen from wastewater

Category	Seaweed type	Wastewater source	Treatment conditions	Contaminants	Treatment efficacy	References
Red seaweed	*Gracilaria lemaneiformis*	Aquaculture	Cage co-culturing seaweed with the fish *Pseudosciaena crocea* Water salinity: 26–29 pH: 7.43–7.83 Temperature:18.4–26.0 °C Time: 20 days	Phosphate and nitrogen	Nitrogen: 21.0% Phosphate: 28.6%	Wei et al. (2017)
	*Gracilaria chouae*	Aquaculture	Co-culturing seaweed with *Sparus macrocephalus* (black sea bream) Temperature: 16.61–22.68 °C Water salinity: 28.33–31.07 Time: 28 days pH: 8.16–8.2	Phosphate and nitrogen	Nitrogen: 41.2% (nitrate-nitrogen: 37.76%, nitrite-nitrogen: 36.99%, ammonia–nitrogen: 29.27%) Phosphorus: 46.2% (Phosphate–phosphorus: 40.64%)	Wu et al. (2015)
	*Gracilaria tikvahiae*	Shrimp wastewater	Co-culturing seaweed with Litopenaeus *vannamei* (Pacific white shrimp) Temperature: 18–33 °C Salinity: 30.4–34.8 g/kg Time: 18 days pH: 7.4–7.9	Nitrogen	Nitrogen: 35%	Samocha et al. (2015)
	*Agarophyton tenuistipitatum* *Hydropuntia edulis*	Brackish water	Temperature: 27–30 °C Salinity: 20% Seaweed biomass density: 0–4.5 g/litres pH:7.75–8.19 Time: 0-2 h hours	Nitrogen and Phosphorus	Optimal removal at 3.5 g/litres (ammonia–nitrogen > 80% Phosphate phosphorus removal > 20%)	Sarkar et al. (2020)
	*Gracilaria lemaneiformis*	Seawater	Temperature: 20 ± 2 ℃, salinity: 30 ± 0.2, irradiance: 80 micromole/(cubic metre/second) with a photoperiod of 12-h light: 12-h dark. Time: 3 days	Nitrogen and Phosphorus	Ammonia–nitrogen (45.99–59.79%, nitrate-nitrogen (13.10–30.21%), nitrite-nitrogen (12.88–14.11%), and phosphate-phosphorus (27.07–31.49%)	Duan et al. (2019)
	*Gracilariacorticata*	Aqueous solution	Metal concentration: 50 mg/l, pH 5, adsorbent dosage: 104 g/l, temperature: 29.9 °C	Cobalt	Cobalt: 87.8%	Raju et al. (2021)
Brown seaweed	*Turbinaria ornata*	Municipal wastewater	Metal concentration: 99.8 mg/l, mixing speed: 250 rounds per minute, adsorbent dosage: 16.2 g/l	Lead	99.80%	Al-Dhabi and Arasu (2022)
	*Sargassum* sp.	Simulated wastewater	Biomass size: 2.2 millimetre Dosage: 0.1 g Temperature: 30 °C pH: 5 Time: 4-h (nickel (ii) ion) and 6-h (copper (ii) ion	Nickel (ii) ion and copper (ii) ion	Copper (ii) ion: 2.06 mmol/gram nickel (ii) ion: 1.69 mmol/gram	Barquilha et al. (2017)
	*Sargassum sp.*	Synthetic wastewater	Time: 60 min Temperature: 25 °C cadmium (ii) ion Seaweed: 0.5 g Rounds per minute: 150 pH: 4 Metal concentration: 5 mg/l zinc (ii) ion Biomass: 1 g pH: 3 Rounds per minute: 200 Ions concentration: 5 mg/l	Cadmium (ii) ion and zinc (ii) ion	Cadmium (ii) ion: 95.3% zinc (ii) ion: 90.3%	Mahmood et al. (2017)
	*Sargassum filipendula*	Simulated wastewater	Sorbent size: 0.737 mm Sorbent: 2 mg/l Temperature: 25 °C Rounds per minute: 180 Time: 24 h pH: 3.5 Metal concentration: 1 mmol/l	Silver, cadmium, chromium, copper, nickel, lead, and zinc ions	Silver: 33.62% Cadmium: 78.03% Chromium: 72.8% Copper: 69.05% Nickel: 32.74% Lead: 56.19% Zinc: 44.21%	Cardoso et al. (2017)
	*Sargassum dentifolium*	Simulated wastewater	Sorbent dosage: 1.5 g/ 100 ml, Ion concentration: 100 parts per million, the flocculation contact time was 1 h followed by 12 h static, temperature: 50 °C, pH: 7	Chromium	Chromium (VI): 99.68%	Husien et al. (2019)
	*Cystoseira crinite and Cystoseira barbata*	Aqueous and wastewater solutions	Chromium (III): Dosage: 100 parts per million, pH 4.5, contact time: 120 min, adsorbent dosage: 0.1 g/50 ml. Chromium (VI): Ion concentration: 100 parts per million, adsorbent dosage: 100 mg/50 ml, contact time: 24-h, pH: 2.0	Chromium (III) and Chromium (VI)	Chromium (III): 73.34% (*Cystoseira crinite*), 70.70% (*Cystoseira barbata*) Chromium (VI): 28% (*Cystoseira crinite*) 35%, (*Cystoseira barbata*)	Yalcin and Ozyurek (2018)
	*Seaweeds* *Ulva intestinalis, Gracilaria sp.,* *Fucus spiralis, Osmundea pinnatifida, Ulva lactuca, Fucus vesiculosus*	Synthetic seawater	Metal concentration: 1 micromole per cubic decimetre, 72 h contact time, temperature: 22 °C, pH: 8.5, salinity of 30 g per cubic decimetre	Mercury	95% 90% 85% 80% 90% 80%	Fabre et al. (2020)
	*Sargassum muticum*	Mining-influenced water	Metal dosage: 2.5 mg/l, temperature: 293 Kelvin, pH: 7	Arsenic	Almost 100%	Vieira et al. (2017)
	*Caulerpa scalpelliformis*	industrial wastewater	Adsorbent dosage: 1.5 g/litre, temperature: 30 °C contact time: 1 h, pH: 5.7 Agitation: 150 rounds per minute	Zinc	83.3 mg/gram	Jayakumar et al. (2021)
	*Sargassum polycystum*	Simulated water	Cadmium: Adsorbent dosage: 1.8 g/l, pH: 4.65, agitation speed: 76 rounds per minute Zinc: pH: 5.7, agitation speed: 125 rounds per minute. adsorbent dosage: 1.2 g/l	Multi-metals	Cadmium: 86.20 Zinc: 92.90%	Jayakumar et al. (2022)
	*Sargassum filipendula*	Real and synthetic effluents	Metal concentration: 1 mmol/l (19.56 mg/l of nickel; 17.33 mg/l of chromium; and 21.79 mg/l of zinc) Temperature: 50 °C	Multi-metals	Chromium: 0.864 mol/g Zinc: 0.302 mmol/g Nickel: 0.347 mmol/g	Costa et al. (2020)
Green seaweed	*Ulva rigida*	Simulated wastewater	Temperature: 20 °C Sorbent: 0.5 g Sorbent size: 0.5 cm Time: 5 h Rounds per minute: 180 Metals concentration: 25 mg/l	arsenic cation (3 +), arsenic cation (5 +), antimony (3 +) selenium (4 +) ions and selenium (6 +) ions	Selenium: (4 +) ions: 0.5 mg/g (pH: 2–4) Selenium: (6 +) ions: 0.2 mg/gram (pH: 2–3) Limited removal efficiency towards arsenic but was effective for antimony and selenium	Filote et al. (2017)

Seaweeds demonstrated a great capacity for removing nutrients from wastewater. For instance, seaweeds can absorb over 41% of nitrogen and phosphorus. In addition to nitrate-nitrogen, ammonia–nitrogen, and nitrite-nitrogen, seaweeds can also remove other nitrogen forms. Similarly, seaweeds can absorb a variety of heavy metals, including copper, cobalt, iron, zinc, lead, nickel, cadmium, silver, chromium, arsenic, antimony, and mercury. This makes seaweeds a very promising option for wastewater treatment on a large scale

In addition to heavy metals and nutrients, seaweeds can absorb other pollutants. For instance, Navarro et al. (2008) examined the sorption of phenol compounds by the *Macrocystis integrifolia* and *Lessonia nigrescens* seaweeds. Findings revealed the highest sorption efficacy of 35% at pH 10 by *Macrocystis integrifolia* due to a completely polar sorption pathway alongside an electrostatic sorption process. This study emphasised that phenol adsorption onto the seaweed’s surface has occurred through the interaction of hydrogen bonds with the hydroxyl groups of the seaweed’s polysaccharides, such as alginates. Common aromatic hydrocarbons were studied by applying red, green, brown, and seaweed biomass to toluene and benzene biosorption (Flores-Chaparro et al. 2017). Results demonstrated that *Phaeophytes* could remove toluene and benzene by 28 and 112 mg/gram, respectively. The sorption process was ascribed to hydrophobic interaction mostly with lipids and, to some extent, with carbohydrates and proteins through nonspecific Van der Waals relations.

In addition, the bioaccumulation of micropollutants by freshwater seaweed has been demonstrated to be a crucial method for removing sulfamethoxazole, triclosan, and trimethoprim (Bai and Acharya 2017). The intracellular seaweed biodegradation is found to be the most useful biosorption approach by which seaweed cells may remove chemical contaminants from the environment (Xiong et al. 2018). In this context, nearly 30–80% of hazardous chemicals, including ibuprofen, tris(2-chloroethyl) phosphate, carbamazepine, and caffeine in wastewater, were biodegraded within the seaweed’s cells (Matamoros et al. 2016; Hom-Diaz et al. 2017; Ding et al. 2017). Thus, the sorbent properties of seaweeds can be viewed as a viable option for reducing the toxic impact of multiple contaminants in aquatic environments, which is favourable for combined energy production.

### Summary

Seaweed can act as a carbon sink by storing seaweed particles in the deep ocean or drifting them in sediments. In addition, other carbon sequestration pathways of seaweeds farming, such as biofuel production that mitigates carbon dioxide emissions and replaces fossil fuels, acting as biofertilisers that replace synthetic fertiliser, lowering methane emissions when used as cattle feed, inhibiting water wave energy, and protecting shorelines that mitigate climate change, increasing water pH and providing oxygen to the waters that decrease ocean deoxygenation and acidification. Consequently, seaweeds contribute to carbon sequestration, coastal safety, carbon sink, food security, and the control of ocean deoxygenation and acidification; therefore, seaweed is remarkably regarded as a promising blue carbon adaptation and climate change mitigation strategy.

Seaweeds can remove pollutants and nutrients from wastewater, transforming waste into valuable commodities. Currently, seaweed cultivation is used for plutonium/uranium removal and refining wastewater runoff. Pollutants can be mitigated by growing seaweed on industrial discharges.

## Seaweed as a feedstock for the anaerobic digestion process

Increasing global energy demands and the negative environmental impacts of fossil fuels increase the need for sustainable and eco-friendly biofuels. Seaweeds can be converted into high-value products, such as biofuels; consequently, they are considered promising third-generation feedstocks in bioremediation (Wang et al. 2020a). By 2054, biofuels derived from seaweed can replace the demand for fossil fuels in the transportation sector, thereby reducing greenhouse gas emissions (Lehahn et al. 2016). As previously discussed in Sect. 2.2, the ability of seaweeds to produce biogas can be attributed to their overall structure.

Utilising thermochemical conversion, anaerobic digestion, and fermentation, seaweed feedstocks were converted into biofuels (Rajak et al. 2020; Wang et al. 2021a). Thermochemical conversion and fermentation are energy-intensive processes that necessitate dehydration and dewatering (Wang et al. 2020a). However, by utilising seaweed for biogas production, all seaweed components, including carbohydrates, lipids, and protein, can be utilised without dehydration, thereby avoiding energy need (Thakur et al. 2022). Due to seaweed’s inexpensive polysaccharides and low lignin content, seaweed is promising biomass for the anaerobic digestion (Farghali et al. 2021). In addition, growing concerns about the depletion of fossil fuels and the increase in greenhouse gas emissions have necessitated the investigation of alternative resources for bioenergy production (Rajak et al. 2020). In this context, seaweed is considered third-generation biomass for bioenergy generation via anaerobic digestion, and seaweed can overcome the inherent limitations of using first- and second-generation feedstock (Ap et al. 2021).

**Fig. 4:**
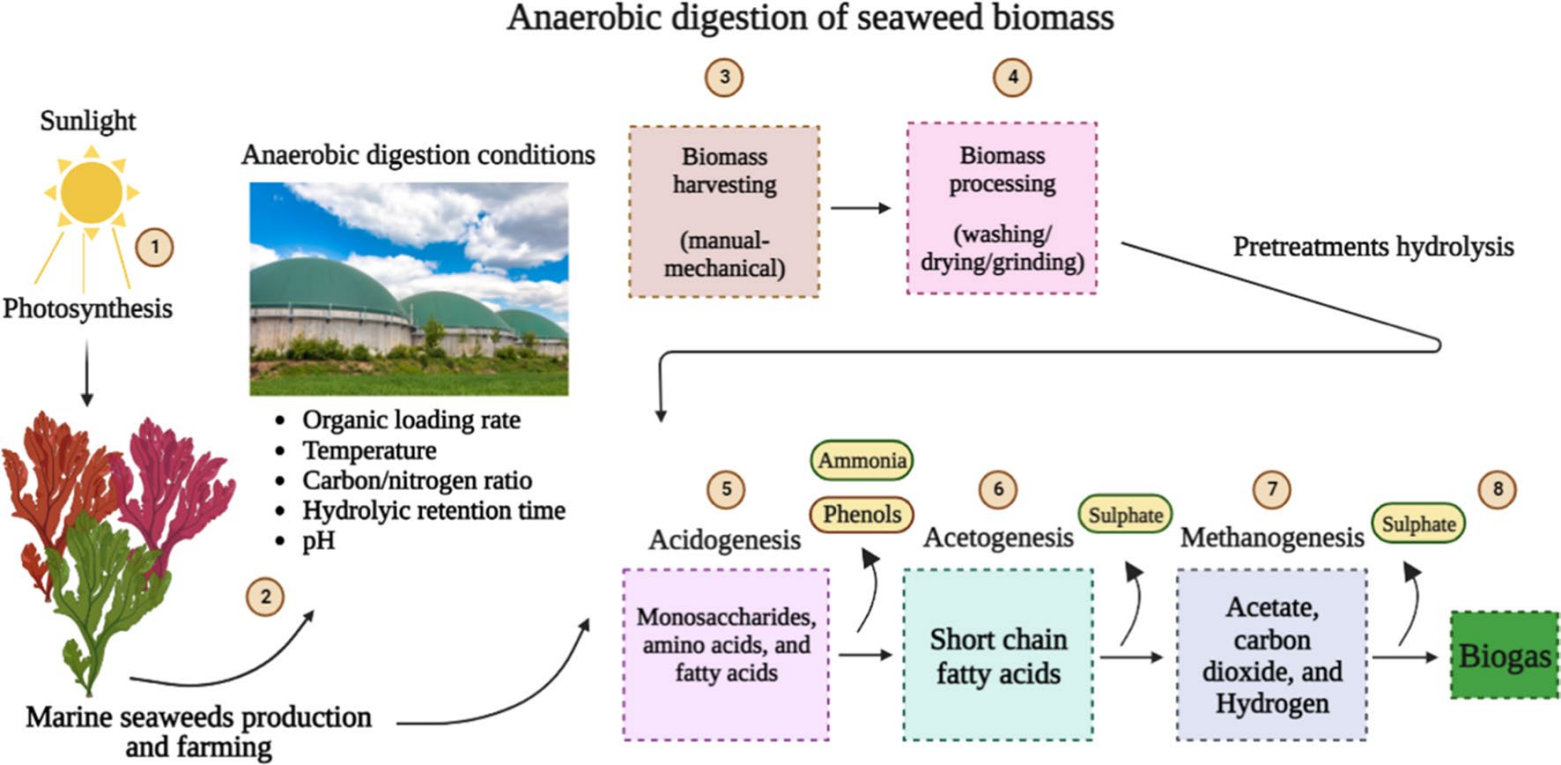
Biogas production from seaweed resources: the mass of wild seaweed grown in aquatic water or farmed seaweed can be gathered manually or mechanically. After assembly, the seaweeds are managed, including rinsing with water, and then the dried or wet biomass is utilised for methane production. In the biogas digester, biomass undergoes four phases of anaerobic digestion, namely hydrolysis, acetogenesis, acidogenesis, and methanogenesis, in order to produce methane and carbon dioxides as end products. Diverse inhibitors and process parameters, such as ammonia, sulphates, phenols, organic loading rates, hydraulic retention time, and other factors, may affect the biogas yields from seaweed feedstocks

Hydrolysis of seaweed biomass generates volatile fatty acids and promotes the production of methane (Fig. 4). The generation of biogas from seaweed has not been thoroughly evaluated. The available reviews lack an understanding of the primary obstacles that limit methane production from seaweed feedstock, as well as the various methods that have been implemented to increase the biogas yield and suggest full utilisation of biomass.

Diverse biogas yields have resulted from the anaerobic digestion of seaweed due to species diversity and seasonal variation in the chemical characteristics of the biomass (Milledge et al. 2019), with brown seaweed digestion yielding comparatively larger methane than that from green seaweeds (Sutherland and Varela, 2014). Even though biochemical batch tests demonstrate inconsistency in reported biogas yields, seaweed as biomass for biogas production has the potential to be an economically viable marine biomass when considered in the context of the circular economy (Milledge et al. 2019). Baltrenas and Misevicius (2015) examined the biogas potential of three seaweeds, *Cladophora glomerata*, *Chara globularis*, and *Spirogyra neglecta,* under mesophilic conditions (35 ± 1 °C). The results illustrated that *Spirogyra neglecta* and *Cladophora glomerata* produced 0.23 and 0.20 cubic metres of biogas per cubic metre of biomass per day, respectively, with biomethane contents exceeding 60%.

Biomethane production from green seaweed *Ulva lactuca* was evaluated in batch experiments after ulvan, protein, and sap extraction with individual and sequential extraction methods. Both treatments enhanced methane yields with the highest biomethane yield of 408-ml methane/gram volatile solids added from sap and ulvan residues (Mhatre et al. 2019). Anaerobic co-digestion of Mediterranean Sea *Ulva* *rigida* generated 408 ml of biogas when mixed with anaerobic sludge (Karray et al. 2017a). Allen et al. (2015) reported that the biomethane potential of cast brown seaweed was 342 and 166 L methane per kilogram of volatile solid for *Saccharina latissimi* and *Ascophyllum* *nodosum*, respectively. Nearly 30 megatonnes of wet shore seaweed are collected annually in Ireland, referred to as the wild harvest. Compared to the average biomethane price of 0.2 € per cubic metre, the anaerobic digestion of Irish seaweed resources combined with cattle slurry, food waste, and grass resulted in a financial incentive of 0.85–1.17 € per cubic metre (Rajendran et al. 2019). Washed and macerated *Gracilaria* *vermiculophylla* was anaerobically co-digested with 2% glycerol and 85% sewage sludge and produced 599 and 605 L of methane per kilogram volatile solid, respectively (Oliveira et al. 2014). Under mesophilic batch anaerobic digestion (38 °C), Ap et al. (2021) found that the biomethane yield of *Sargassum fulvellum* seaweed was 142.91-ml methane per gram volatile solid for macerated biomass (75–850 µm) compared to 68.11-ml methane per gram volatile solid for the raw biomass (106 µm–4.75 mm). However, under thermophilic batch digestion mode (55 °C), Farghali et al. (2021) found that the same untreated raw seaweed produced 145.69 ml of methane per gram of volatile solid. Nevertheless, the operational conditions, such as temperature and pretreatment method before anaerobic digestion, have the potential to influence biogas production. Consequently, the subsequent section discusses the difficulties of biogas production from seaweed and the potential solutions.

### Challenges of biogas production from seaweed

#### Seaweed cell wall rigidity

The anaerobic digestion of seaweed is limited by the firmness of the cell wall and the complexity of the biomolecular organic structures in seaweeds, which inhibits the fragmentation of the recalcitrant cell wall during hydrolysis and prolongs the anaerobic fermentation time (McKennedy and Sherlock 2015). The primary structural cell wall component in brown seaweed is cellulose, while in red and green seaweed, the primary structural cell wall component is cellulose, xylan, mannan, and xylan. These polysaccharides form various configurational microfibril structures, including flat ribbons in the case of cellulose and mannans and a helix configuration in the case of xylans (Maneein et al. 2018). Depending on the species, microfibrils with variable orientations are typically linked to polysaccharides matrix to form various carboxylic or sulphated polysaccharides (Synytsya et al. 2015). For example, sulphated fucans extracted from brown seaweed *Himanthalia elongate* have been suggested to interlock the cellulosic structure, whereas alginate–phenol bonds are the primary linkage governing the rigidity of seaweed cell walls (Deniaud-Bouet et al. 2014; Tiwari and Troy 2015). The association between protein in brown seaweed and phenols and sulphated fucans was observed (Deniaud-Bouet et al. 2014).

Moreover, phenols may have inhibitory effects on the anaerobic microorganisms (Maneein et al. 2018). Ulvans present in green seaweeds, including xylose, galactose, uronic, and rhamnose acid, are comparatively resistant to biodegradation and might constrain access to the disintegration of other polysaccharides, particularly starch and cellulose (Maneein et al. 2018). Therefore, the structural rigidity of seaweed's cell wall architecture, which is dominated by alginates and sulphated fucans in brown seaweeds, agar and carrageenans in red seaweed, and ulvans in green seaweed, prevents seaweed from being hydrolysed by microbes to monomers (glucose) (Maneein et al. 2018). Ometto et al. (2018) ascribed the low specific methane yield of *Saccharina latissima* seaweed to the high contents of alginate and lower level of readily biodegradable laminarin and mannitol. The rate-limiting phase of the anaerobic digestion of seaweed is considered to be the hydrolysis of complex polysaccharides. Therefore, partial removal of complex polysaccharides enhanced the biodegradability of seaweed in the actual fermentation reactor (Tedesco and Daniels 2018). Polyphenols and insoluble fibres have also been identified as hardly biodegradable and potential anaerobic digestion inhibitors (Jard et al. 2013). In addition, seaweed’s crystalline structure, surface properties, cellulosic polymers, lignin content, fibre strength, and the presence of hemicellulose materials are listed as other factors that influence the biodegradability of seaweed (Tedesco and Daniels 2018).

The biodegradability index quantifies the methane potential of biomass in relation to biomass’s theoretical biomethane yield. The obtained value indicates the degree of substrate biodegradation and biomethane yield relative to the theoretical yield of methane (Allen et al. 2015). Table 6 shows various biodegradability indexes for some seaweeds. *Saccharina latissima* showed the highest degradability index of 0.81. Anaerobically biodegradability index of *Fucus serratus* and *Ascophyllum nodosum* is 0.19–0.34 for 30 days; in addition, 66–81% of their volatile solid contents were not biodegraded due to high lignocellulose content (Lin et al. 2019). Overall, *Sargassum* brown seaweed is less biodegradable by anaerobic digestion than *Ulva* green seaweed and *Gracilaria* red seaweed (Maneein et al. 2018). Higher insoluble fibre values can support this in brown seaweed (10–75%) compared to green seaweed (29–67%) or red seaweed (10–59%) (Maneein et al. 2018; Cabrita et al. 2017). Accordingly, pre-treatment techniques have been suggested based on the type and structural composition of the seaweed, as described in the following section.

**Table 6:** Biomethane potential and biodegradability index of some seaweeds (Allen et al. 2015; Tabassum et al. 2017a)

Seaweed biomass	Methane production (litre methane/kilogram volatile solid)	Theoretical biomethane yield (litre methane/kilogram volatile solid)	Biodegradability index	Methane potential (cubic metre methane/tonne of wet weight)
*Saccharina latissima*	342	422	0.81	34.5
*Fucus spiralis*	235	540	0.44	32.7
*Saccorhiza polyschides*	263	386	0.68	34.5
*Alaria esculenta*	226	474	0.48	26.9
*Ascophyllum nodosum*	166	488	0.34	32.3
*Himanthalia elongate*	260	334	0.78	21.1
*Ulva lactuca*	190	465	0.41	20.9
*Laminaria digitata*	218	479	0.46	22.5
*Fucus serratus*	101	532	0.19	13.5
*Fucus vesiclosus*	126	249	0.51	19.4

The biodegradability indices of seaweeds ranged from 0.19 to 0.81. The presence of recalcitrant components that are difficult for anaerobic microbes to digest is responsible for these wide variations. The presence of recalcitrant substance within the structure of seaweed resulted in methane production ranging from 101 to 342 L/kg of volatile solid, whereas the theoretical methane potential ranges from 249 to 540 L/kg of volatile solid

#### Effect of seasonal variations

Seasonal and geographical variations in the carbohydrate composition of seaweeds reduce the methane recovery from seaweed (i Losada et al. 2020). For instance, harvesting Irish seaweed during different seasons altered the seaweed's physicochemical properties, chemical composition, and subsequent methane yield. Tabassum et al. (2016a) found that *Laminaria digitata* seaweed biomass harvest was 4.5 folds higher in August compared to that in December, with biomethane production 1.4 times higher in August (327 L methane per kilogram of volatile solid). Additionally, Tabassum et al. (2016b) found that *Ascophyllum nodosum* Irish seaweed collected in the summer season had a higher polyphenolic value than that harvested in October. Therefore, specific methane yield was 2.9 times (47 cubic metres of methane per tonne wet weight) higher in October compared to the seaweed collected in December.

The *Laminaria* spp. seaweed harvested in November produced 342 L of methane per kilogram of volatile solid, whereas the same seaweed collected in March produced 163 L of methane per kilogram of volatile solid (Montingelli et al. 2016a). Maneein et al. (2021) examined the biogas production from *Sargassum muticum*. They found a high methane yield from *Sargassum muticum* collected in *s*pring with a value of 19.7 L methane per kilogram wet weight over those harvested in summer, which showed 13.0 L methane per kilogram wet weight. The rapid methane production rate from spring-harvested seaweed was attributed to the increased availability of biodegradable carbohydrates, such as mannitol, which were readily bioconverted to methane. In addition, this variation was attributed to the fact that summer-harvested seaweed contained polyphenolics that were 3.8 times higher than spring-harvested seaweed.

The seasonal variation effects on the seaweed’s heavy metals content were analysed in *Fucus vesiculosus, Ascophyllum nodosum, Alaria esculenta,* and *Saccharina latissima,* which were collected in four various seasons (summer, spring, winter, and autumn). Generally, the contents of phosphorus, potassium, sodium, calcium, aluminium, magnesium, iron, and sulphur were higher during summer and spring. During the winter and autumn, however, only arsenic levels were higher (Ometto et al. 2018). Table 7 outlines the effects of different seaweed harvesting seasons on biomethane yield.

**Table 7:** Seasonal influence on the chemical composition and methane production of seaweed

Seaweeds	Harvesting month	Total solid (%)	Volatile solids (%)	Carbon content (%)	Nitrogen (%)	Hydrogen (%)	Carbon/nitrogen ratio	Ash (%)	Theoretical biomethane potential (litres methane per kilogram volatile solid)	Biomethane potential (litres methane per kilogram volatile solid)	Biomass conversion (%)	Remarks	References
*Laminaria digitata*	August	19.72	16.12	36.76	1.14	5.54	32.24	18.28	452	327	72.34	High carbon content and biomethanation were observed in August	Tabassum et al. (2016a)
	January	11.43	7	26.06	3.95	3.38	6.59	38.82	421	237	56.29		
*Ascophyllum nodosum*	April	20.99	15.94	36.14	2.47	5.03	14.63	24.06	497	217	43.66	High polyphenolic compounds resulted in a low methane production	Tabassum et al. (2016b)
	October	28.52	22.01	40.67	0.89	5.11	46.75	22.83	543	215	38.59		
*Laminaria digitata* (dry biomass)	July	93.7	65	30.8	1.4	5	22	Not mentioned	366	293	80.05	High biomethane production resulted from high organic content	Membere and Sallis (2018)
*Saccharina latissima*													
(dry biomass)	June	99.9	66	31.5	2.2	4	14.3	Not mentioned	443.9	281.4	63.3	Low carbon/nitrogen ratio lowered the methane yield	Lin et al. (2019)
*Ulva lactuca*	July	18.03	10.88	30	3.5	4.4	8.5	39.7	465	190.1	40.88	Reduced methane was noted owing to elevated salinity	Tabassum et al. (2017a)
*Fucus vesiculosus*	July	21.28	16.11	26.8	1.5	3.2	17.6	24	249	126.3	50.7	Less methane yields due to the occurrence of inhibitors	Allen et al. (2015)
*Saccorhiza polyschides*	July	15.25	13.11	36.1	1.6	5	23.2	14	386	263.3	68.21	High carbon quantity improved methanation	
*Fucus spiralis*	July	19.72	13.92	36.1	2.1	4.7	17.3	29.4	540	235	43.51	High recalcitrant components reduced the methane generation	Tabassum et al. (2017a)
*Laminaria digitata*	March	9.74	6.49	30.41	3.70	3.97	8.22	33.33	469	245	52.23	Low carbon/nitrogen ratio reduced the methane generation	Tabassum et al. (2017b)
*Ulva intestinalis*	Summer	18.2	14.2	31.5	2.88	4.29	10.9	Not mentioned	598	447.8	74.88	Less sulphate content increased biomethanation	Romagnoli et al. (2019)
*Ulva lactuca*	June	19.12	11.24	25.4	3.3	3.7	7.7	Not mentioned	431	250	58.01	High salinity reduced the biogas yield	Allen et al. (2013)

In terms of carbon, nitrogen, hydrogen, solids, and ash content, seaweeds exhibit seasonal variations in their chemical compositions. Generally, seaweed harvested between September and March (autumn) and July and August (end of summer) is richer in carbohydrates and nutrients, making seaweed more suitable for anaerobic digestion

#### Existence of inhibitory materials

Anaerobic digestion relies on microbial activity to convert complex compounds to monomers, which the microorganisms then consume to produce biomethane (Tawfik et al. 2022b). Typically, seaweed contains polyphenols, sulphated polysaccharides, and halogenated compounds, which inhibit anaerobic microorganisms (Tabassum et al. 2017a). The presence of sulphur-rich biomass in anaerobic digestion led to hydrogen sulphide production by sulphate-reducing bacteria (Farghali et al. 2019). The formation of hydrogen sulphide alongside methane indicates a competition between sulphate-reducing bacteria and methanogens for acetate, resulting in a decrease in methane production (Jung et al. 2022). In addition, the high salt content of seaweed biomass, which included sodium, calcium, potassium, and magnesium salts, led to the accumulation of salts in the anaerobic digestion systems, thereby inhibiting all microbes in anaerobic bioreactors (Maneein et al. 2018). High salinity shifted methanogens from the acetoclastic (*Methanosaeta)* to the hydrogenotrophic methanogens (*Methanocorpusculum* and *Methanobrevibacter*) (De Vrieze et al. 2017). Zhang et al. (2017) found that hydrogenotrophic methanogens (*Methanobacterium)* tolerated salinity up to 85 g/l, whereas acetoclastic methanogens (*Methanosarcina* and *Methanosaeta)* were inhibited at salinity more than 65 g/l during the anaerobic digestion of *Laminaria japonica* seaweed.

In addition, the inhibition of methanogen lowers the pH, leading to the accumulation of volatile fatty acids and the subsequent suppression of the anaerobic digestion process. As part of their chemical defence systems, seaweed also produces a variety of halogenated secondary metabolites, particularly chlorinated and brominated compounds (Nielsen et al. 2020). In 90% of red seaweed and 7% of green seaweed, chlorinated and brominated metabolites predominate, whereas iodine-containing metabolites predominate in brown seaweeds (Nielsen et al. 2020). Some brown seaweed types can build up to 1.2% of the iodine per seaweed dry weight. Halogens are well-known inhibitors of biomethane production from anaerobic digesters (Nielsen et al. 2020). Halogenated compounds inhibited the growth of anaerobic microorganisms. Specifically, halogenated aliphatics inhibited methanogenesis (Czatzkowska et al. 2020), which is frequently produced by seaweed (Leri et al. 2019). *Saccharina latissima* may generate up to 120–630 mg of organochlorine and aliphatic organobromine per kilogram dry weight of seaweed (Czatzkowska et al. 2020).

Algae and marine and terrestrial organisms collectively contain more than 8000 phenolic compounds (Perez et al. 2016). Particularly brown seaweeds contain substantial amounts of phenolics (about 14% dry weight). In many seaweeds, phlorotannins predominate among various polyphenols (Milledge et al. 2019; Montero et al. 2016). Seaweed polyphenol inhibits anaerobic digestion microbiota and reduces biogas production (Milledge et al. 2018; Tabassum et al. 2016c).

#### Insufficient trials and cost

The widespread use of seaweed as biomass for biogas production is still in the infancy stage. At the industrial level, only a handful of nations, including South Korea, Taiwan, and Brazil, have begun to develop seaweed bioenergy projects (González-Gloria et al. 2021). Biogas production from seaweed is unstable, with several variations between species and seasons; in addition, the presence of inhibitory compounds and the recalcitrant characteristics of seaweed necessitate pretreatments for the large-scale application of biogas systems. Washing seaweed and other mechanical or physical pretreatments incur additional costs. Farghali et al. (2021) found that biological and chemical pretreatment of seaweeds resulted in not only higher biomethane yield but also positive energy balance from alkaline and enzymatic pretreatment of seaweeds. However, when the authors estimated the cost of seaweed pretreatment, they could not identify a net profit due to the higher price of enzymatic and chemical additives. Consequently, the macroalgae-based biofuels industry must optimise and develop more research and technologies to reduce costs and equipment (González-Gloria et al. 2021).

### Pre-treatments approaches to overcome seaweed’s challenges

**Fig. 5:**
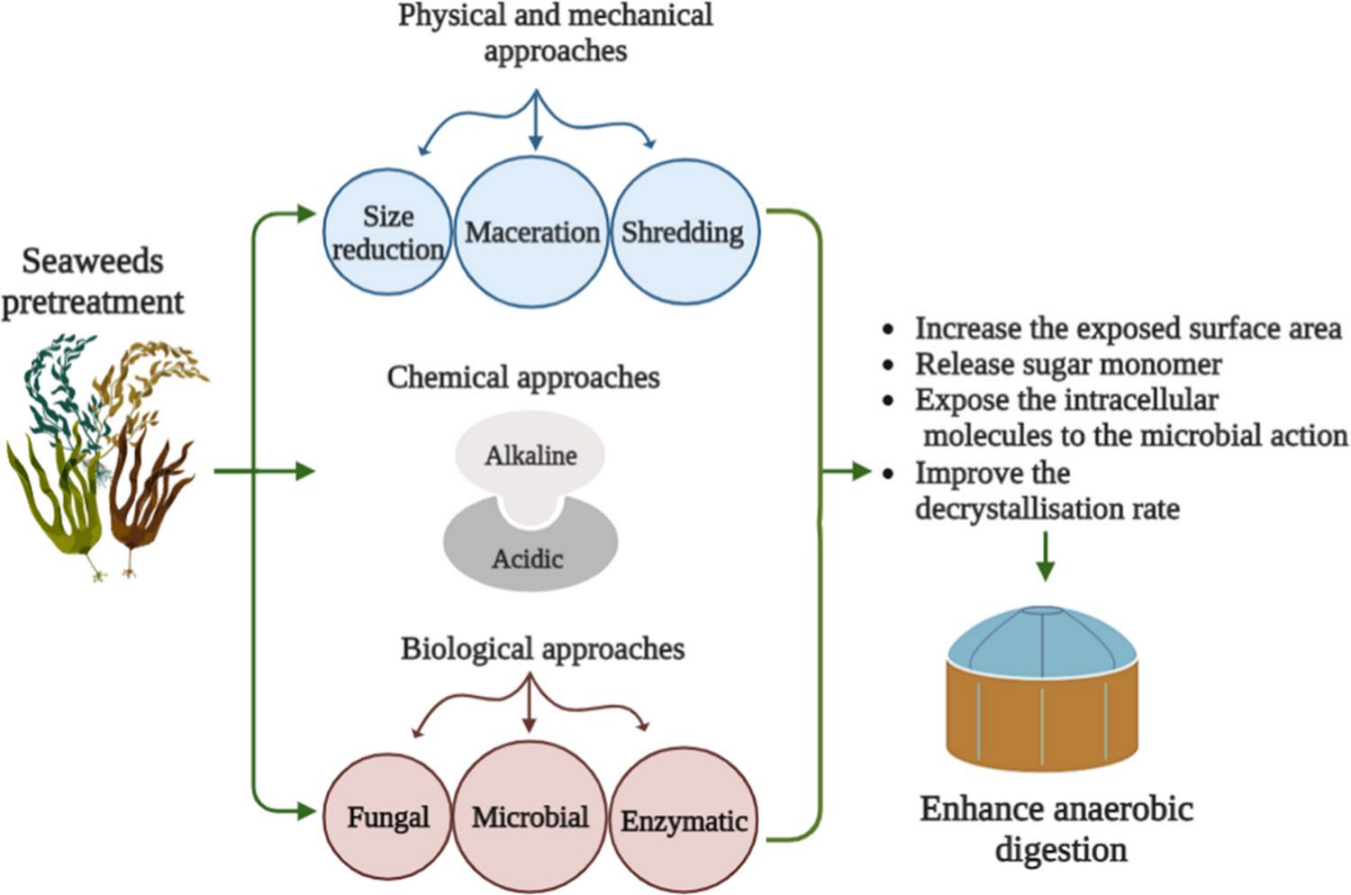
Methods of pretreatment for seaweeds. Various pretreatment methods can be applied to seaweeds, including physical (mechanical), chemical, biological, thermal, and integrated methods. The applied pretreatment increases the exposed surface area, degrades the cell wall, releases sugar monomer, exposes the intracellular molecules to microbial and enzymatic action, and improves the decrystallisation rate, which would be better utilised for methane production, thereby enhancing anaerobic digestion. A method that is both environmentally friendly and cost-effective is still required. Biological pretreatment is an effective and environmentally friendly process

The presence of recalcitrant substances, sulphide, and high salinity reduces the biogas production from seaweed feedstocks, which can be enhanced by a variety of pretreatment methods, including physical, mechanical, chemical, thermal, biological, and integrated methods, as shown in Fig. 5.

Ap et al. (2021) examined the influence of different pretreatments, including chemical, mechanical, and biological, on the mesophilic anaerobic digestion from *Sargassum fulvellum* seaweed. Among different treatments, mechanical pretreatment through maceration of seaweed to 75–850 µm enhanced the biomethane outcome by 52.34% compared to the control seaweed of 106 µm–4.75-mm particle size. Mechanical pretreatment improved cellulose biodegradation rate by approximately 3.4 folds, optimising microbial growth in a decreased-sized seaweed-containing bioreactor. In addition, the mechanical pretreatment of seaweed increases the exposure of intracellular molecules to microbial action, hence improving anaerobic digestion (Ap et al. 2021; Ganesh Saratale et al. 2018). Mechanical pretreatment of *Fucus vesiculosus* (5 mm chopped) followed by batch anaerobic digestion resulted in double methane yields than the untreated biomass (Pastare et al. 2016). However, some authors found that the pretreatment of seaweeds decreased the biogas yield. For instance, maceration of *Laminaria spp*. to 1–2 mm-size particles reduced methane yield by 26.52–20.73% more than raw seaweed (Zheng et al. 2011). Biomass with a small particle size has been more susceptible to agglomerates and under compaction, which results in reduced direct contact between seaweed particles and microorganisms during anaerobic digestion (Farghali et al. 2021). Additionally, excessive maceration could increase the hydrolysis of organic materials, hence generating volatile fatty acids, and inhibiting the methanogens (Zheng et al. 2011). Likewise, the size reduction of brown seaweed *Saccharina latissimi* decreased the biogas yields at thermophilic (53 °C) digester (Montingelli et al. 2016b) due to inadequate membrane disruption of the seaweed through the size reduction.

Apart from mechanical pretreatment, biological pretreatment of seaweed is also investigated using different enzymes and biological agents. Tapia-Tussell et al. (2018) investigated the fungal pretreatment of *Trametes hirsute* on the anaerobic digestion of Mexican Caribbean seaweed and found a 20% increase in methane yield over the control untreated seaweeds. Under thermophilic anaerobic digestion (55 °C), biological treatment of *Sargassum fulvellum* seaweed by adding cellulase enzyme enhanced the biomethane yield to 116.64% compared to the untreated seaweed (Farghali et al. 2021). In addition, chemical pretreatment of *Sargassum fulvellum* with 0.36 ml/g volatile solid and 0.18 ml/g volatile solid of 2 molars hydrochloric acid and with 0.09 ml/g volatile solid and 0.04 ml/g volatile solid of 6 molar sodium hydroxides for 24 h at room temperature boosted the methane yield by 15.11, 6.53, 45.65, and 37.01% compared with the unpretreated control (Farghali et al. 2021). However, the authors demonstrated that the biological pretreatment of seaweed was the most effective method. The enzymatic pretreatment of *Ulva rigida* generated 7.3 g per litre of reduced sugar monomer that resulted in a biogas yield of 626.5 ml/g of chemical oxygen demand compared to only 0.6 g per litre of reduced sugar for the untreated control (Karray et al. 2015). Biological pretreatments changed bacterial and archaeal diversity and boosted biogas production (Zou et al. 2018).

The higher biogas outcome obtained from the biological pretreatment of seaweed was attributed to the rapid cell wall degradation and solubilisation via enzymatic hydrolysis, which enabled the release of the recalcitrant components, including cellulose, and more valuable lipids and sugars monomers in higher quantities to microbial action, which could be more utilised for biomethane generation (Farghali et al. 2021). Biological pretreatment of seaweed is a low energy, promising alternative to other energy-intensive pretreatments, and it does not produce any inhibitory by-products during the anaerobic digestion process; therefore, we recommend additional research on the biological treatment of seaweed.

The solubilisation of hemicellulose, polymers, and lignin by chemical pretreatment facilitates the microbial solubilisation of seaweed. In addition, alkaline pretreatment can cleave and saponify lignin–carbohydrate bonds, increase the internal surface area and porosity and reduce the degree of crystallisation and polymerisation of seaweed, thereby optimising the monomers' accessibility to subsequent microbial digestion (Farghali et al. 2021; Thompson et al. 2019). In contrast, chemical pretreatments of *Sargassum fulvellum* biomass reduced the gas yield by 5.80–19.54% more than the untreated control (Ap et al. 2021).

Hydrothermal pretreatment of *Sargassum* sp., at a severity factor of 3.83 reduced the hydrogen sulphide formation from 3 to 1%, maximised soluble chemical oxygen demand production to 27,250 mg/l more than the unpretreated seaweed (237%), with maximum biomethane yield obtained of 408-ml methane/gram volatile solids (Thompson et al. 2020). Table 8 summarises the overall effect of different pretreatment conditions on biogas yield from various seaweed biomass.

**Table 8:** Pretreatment approach to improve the solubilisation of seaweed feedstocks and enhance biogas generation

Seaweeds	Treatment	Conditions	Anaerobic condition	Biogas/methane production	Major remark	References
*Ulva* sp.	Biological	Fungal fermentation	Mesophilic (35 °C)	153-ml methane/gram volatile solid	Enhanced biogas yield by 21% and allowed an eco-friendly valorisation Biodegradability of 57%	Ben Yahmed et al. (2017)
*Ulva intestinalis*	Integrated (microwave-iron oxide nanoparticles)	Microwave and iron oxide nanoparticles	Mesophilic (37 °C) with continuous shaking at 150 rounds per minute	206-ml biogas/gram volatile solid	Enhanced solubility of organic matter	El Nemr et al. (2021)
*Ulva* sp.	Integrated (thermos-chemical pretreatments with hydrochloric acid or sodium hydroxide)	0.1-mol hydrochloric acid, 90 °C	Mesophilic (35 °C) for 30 days	284.8-ml methane/gram volatile solid compared with 293.0 for thermally treated control (0 hydrochloric acid and sodium hydroxide)	Improvement in solubilisation degree Sodium hydroxide had negative effects on the solubilisation and methanation	Jung et al. (2016)
*Gracilaria manilaensis* & *Gracilariopsis persica*	Thermo-chemical (100 °C, hydrochloric acid)	2 mol hydrochloric acid, 100 °C, 1 h	Mesophilic (32 °C) for 60 days	Both seaweeds produced higher biomethane yields by 70% and 62%, respectively than the theoretical yields 0.281 and 0.237 normal cubic metre/kilogram volatile solid, respectively, compared to 0.191 and 0.148 for the untreated control	Enhanced the biomethane potential of seaweed residues compared with a whole seaweed biomass	Hessami et al. (2019)
*Pelvetia canaliculata*	Mechanical	Hollander beating, 580 rounds per minute, 60 min at a food/inoculum ratio of 0.3	Mesophilic (37 °C)	340-ml methane/gram volatile solid	74% enhanced biomethane production	Rodriguez et al. (2018)
*Laminaria digitata*	Biological	Cellulase for 24 h, Temperature 37 °C at 300 rounds per minute	Mesophilic (35 °C) for 32 days	232-ml biogas/gram volatile solid	2% increased biogas caused by easily solubilised cellulose	Vanegas et al. (2015), Vanegas et al. (2015)
	Acid pretreatment	2.5% citric acid		237 ml biogas/gram volatile solid	6%	
Mexican Caribbean seaweed	Biological	*Trametes hirsuta* fungi for 6 days Temperature 35 °C	Mesophilic (38 °C) for 29 days	104-ml methane/gram volatile solid	20% enhanced biogas yield due to readily biodegradation of cell walls	Tapia-Tussell et al. (2018)
*Sargassum fulvellum*	Biological + chemical	Cellulase enzyme, Acid (hydrochloric acid, 0.18–0.36 ml/g volatile solid) Alkaline (sodium hydroxide 0.04–0.04 ml/g volatile solid)	Mesophilic, 55 °C for 40 days	341.94 ml/g volatile solid for biological pretreated seaweed 242.45–254.46 and 323.77–339.64 ml/g volatile solid biogas, for acidic and alkaline treated seaweed, respectively	116.64% increase in methane yield increase in methane yield by 6.53–15.11% and 37.01–45.65%, respectively	Farghali et al. (2021)
*Sargassum fulvellum*	Mechanical pretreatment	Seaweed milling to 75–850 µm	Mesophilic, 38 °C for 25 days	142.91-ml methane/gram volatile solid	52.34% increase in methane yield	Ap et al. (2021)
*Fucus vesiculosus*	Mechanical	Washed, chopped (5 mm)	Mesophilic, 37 °C for 21–25 days	133.7-ml methane/gram volatile solid	94.9% improved biogas due to the high breakdown of organic materials for microbes	Pastare et al. (2016)
*Laminaria* sp.	Mechanical	Hollander beater, 580 rounds per minute, 15 min	Mesophilic, 38 °C for 14 days	240-ml methane/gram volatile solid	8.6% improvement in methane production with the accelerated digestion process	Montingelli et al. (2017)
*Saccharina latissima*	Hydrothermal + milling to 5 mm	140 °C, 20 min	Mesophilic, 35 °C for 10 days	345.1-ml methane/gram volatile solid	22.64% increase in methane yield Considerable degradation of the recalcitrant seaweeds’ components	Lin et al. (2019)
*Sargassum* sp.	Hydrothermal	Severity factor of 2.65	Mesophilic, 35 °C for 21 days	116.7-ml methane/gram volatile solid	265% greater methane than the raw biomass Reduced concentration of hydrogen sulphide from 3 to 1% in biogas production high arsenic content in digestate	Thompson et al. (2020)
*Fucus vesiculosus*	Combined	Carbon dioxide at 30–100 bar pressure and autoclaving	Mesophilic, 37 °C for 30 days	227.6-ml methane/gram volatile solid	132.5% increased increase in methane yield	Gruduls et al. (2018)
*Fucus lumbricalis*				375.5-ml methane/gram volatile solid	116.4% increase in methane yield	
*Pilaiella littoralis, Ectocarpus siliculosus and Zostera marina*	Combined	Hydrothermal-acidic pretreatment (80 °C for 24 h + 0.15 mol hydrochloric acid)	Mesophilic, 37 °C for 35 days	188.68-ml methane/gram volatile solid	78% higher methane than the raw seaweed Combined pretreatments increased sugar recovery and methane production	Lymperatou et al. (2022)
		Alkaline pretreatment (15% aqueous ammonia for 4 days)		144.16-ml methane/gram volatile solid	36% increase in the methane yield	
*Gracilaria verrucosa*	Washing	Tap water washing for 10 min	Mesophilic, 37 °C for 28 days	92-ml methane/gram volatile solid	33.11% increase in methane yield	Suhartini et al. (2022)
Pelagic *Sargassum*	Steam explosion and extrusion	Streaming at 220 °C with 0.98 megapascals for 24 min Extrusion with 0.3 kg/hour at 200, 220, and 240 °C	Mesophilic, 36 °C for 39 days	184- and 187-ml methane/gram volatile solid, respectively	76.92–79.81% increase in methane yield, respectively The extrusion process was favoured	Ayala-Mercado et al. (2022)
Cladophora* sp. Ulva intestinalis*	Co-digestion	Co-digestion ratio between seaweed and wheat straw was 1:1 (volatile solid basis)	Mesophilic, 37 °C for 30 days	504.5 and 375.8-ml methane/kilogram volatile solid, respectively	Enhanced degradation kinetics and microbial synergism	Romagnoli et al. (2019)
*Ulva rigida*	Co-digestion	1:1 based on volatile solids *Ulva* and Sugar wastewater	Mesophilic, anaerobic up-flow bioreactor, 37 °C for 120 days	Maximum methane production was 114-ml methane/gram volatile solid with 75% of methane	Allowed the recovery of essential nutrients	Karray et al. (2017b)
*Ul*va *lactuca*	Co-digestion	10% seaweed with rumen fluid	Mesophilic, 38 °C for 18 days, 160 rounds per minute	85.6% increased Biochemical methane potential	Boosted the archaeal diversity by rumen fluid	Zou et al. (2018)
*Laminaria digitata*	Co-digestion	80% seaweed: 20% cattle manure	Continuous thermophilic (55 °C), organic loading rate of 2 g volatile solids/litre/day and hydraulic retention time of 15 days	290-ml methane/gram volatile solid	Enhanced biogas yield and buffering capacity	Sun et al. (2019)
*Pelagic Sargassum* sp.	Co-digestion + hydrothermal treatment	25% *Sargassum*:75% food waste Hydrothermal pretreatment: 140 °C (severity factor of 2.65)	Mesophilic, 35 °C for 21 days,	292.18-ml methane/gram volatile solid	Rearranged metal components and increased the buffering capability of the biodigester Solid digestate requires arsenic remediation to fit with standard international guidelines for soil	Thompson et al. (2021)

Several pretreatment strategies can be employed to expedite the breakdown of recalcitrant biomass into monomers that are readily utilised by microbes in anaerobic digesters and then converted into methane. Some treatments increased methane production and biomass decomposition, whereas others, particularly chemical treatments, yielded variable outcomes, as chemical residues can inhibit anaerobic microorganisms

Co-digestion of seaweed with other biomass can eliminate the difficulties of seaweed mono-digestion, where co-digestion balanced the carbon/nitrogen ratio, removed salt accumulation, improved process stability, reduced volatile fatty acid accumulation, provided high nutrient value, increased synergistic influences, and improved digestibility in bioreactors (Karki et al. 2021). A high carbon-to-nitrogen ratio in seaweed biomass reduces the bioavailability of nutrients to anaerobic microorganisms. Co-digestion with high-nitrogen feedstock can overcome nutrient limitations and increase biogas production. Several studies indicated that biomass, such as rice straw, sewage sludge, wastewater, dairy manure, and food waste, could be co-digested with seaweeds (Table 8).

Anaerobic co-digestion of *Laminaria digitata* with dairy manure at 80:20 ratios on volatile solids produced 290-ml methane/gram volatile solid under organic loading rate of 2 g volatile solids/litre/day and 15 days hydraulic retention time and improved the process stability (Sun et al. 2019). *Ulva* seaweed co-digested with cow dung at a 3:1 ratio yielded gas of 574-ml methane/gram volatile solid (Akila et al. 2019). The digestate produced after the conversion of *Ulva* sp. was applied as an alternative to traditional synthetic fertiliser. Similarly, co-digestion of *Cladophora* and *Ulva intestinalis* with wheat straw resulted in high gas outcomes of 504.5 and 375.8-ml methane/gram volatile solid, respectively (Romagnoli et al. 2019). However, pretreatments of seaweeds present some issues, as shown in Table 9; for instance, the high cost and energy intensity are limiting factors in the use of pretreatments for methane enhancements from seaweeds; thus, other promising methods may be required.

**Table 9:** Seaweed pretreatment methods

Method	Condition	Advantage	Disadvantage
Chemical	Sodium hydroxide Hydrochloric acid Citric acid	Solvation and saponification of biomass	Alter the reactor pH Formation of furfurals and phenols
Mechanical	Beating Chopping Ball milling Ultrasonication	Increases reaction surface to volume ratio Release monomers from complex feedstock	Energy-intensive A high hydrolysis rate may inhibit the anaerobic digestion process
Thermal	Heating Autoclaving Hydrothermal Microwave	Break the hydrogen ponds Disrupt structural components of seaweed	Higher reaction temperature (more than 180 °C) may produce phenols, furfural, and furan derivatives
Biological	Enzymes Microbes	Microbes excrete enzymes to solubilise the biomass and promote hydrolysis of seaweed	High cost Need specific conditions and microbes
Integrated approach	Thermo-chemical Mechanico-chemical	Combined mechanisms to optimise the biomass hydrolysis	Complex Energy-intensive methods Requires optimisation

Each pretreatment method has benefits and drawbacks. Chemical and thermal pretreatments may generate furfurals and phenols that inhibit anaerobic microorganisms, thereby inhibiting methane production. The mechanical and integrated processes are energy intensive. If the price of biological agents could be reduced, biological could be a promising process (Thakur et al. 2022)

### Summary

Seaweeds can be converted into biofuels by providing a steady feedstock supply for anaerobic digestion. Biogas production from seaweed is still hindered by numerous obstacles, such as the recalcitrance of seaweeds, seasonal biomass variation, the presence of inhibitory compounds, and the expense of harvesting. Several pretreatment methods, including chemical, mechanical, biological, thermal, and co-digestion, have been utilised by researchers to combat and improve the efficiency of anaerobic digestion and overcome seaweed limitations. Higher methane yield after pretreatment was attributed to a greater quantity of the released organics from the chemically pretreated seaweed, which were rapidly utilised during the early digestion stage and favoured the methanogenic consortium. However, the large-scale and cost-effective application is lacking. In addition, urgently required are cost analyses and life cycle assessments of seaweed's anaerobic digestion. The study of microbial shifts following pretreatments is an intriguing area for future research. A comprehensive evaluation of microorganisms would provide a detailed understanding of inhibitory pathways and strategies that can be implemented to increase methane yields. The labour-intensive collection of seaweed necessitates the incorporation of more bioengineering-based tools.

## Use of seaweed for biochar production

### Seaweed biochar

In an oxygen-free environment, char is produced by converting biomass to a carbon-rich black material via thermal processes such as pyrolysis and/or gasification (biochar) or hydrothermal carbonisation (hydrochar) (Farghali et al. 2022a; Farghali et al. 2022b; Mona et al. 2021; Singh et al. 2021). The thermochemical processes permanently alter the physicochemical structure of biomass (Osman et al. 2022). Table 10 summarises the biochar produced by diverse seaweeds through various thermochemical processes.

**Table 10:** Various biochar yields from diverse algal species utilising thermochemical treatments

Seaweed	Char producing condition	Temperature (°C)	Biochar yield (% wet weight)	Application	References
*Spirulina*	Slow pyrolysis	900	25.96	Soil amendment, biofuel	Chaiwong et al. (2012)
*Spirogyra*	Slow pyrolysis	900	18.61	Soil amendment, biofuel	Chaiwong et al. (2012)
*Cladophora*	Slow pyrolysis	900	32.21	Soil amendment, biofuel	Chaiwong et al. (2012)
*Arthrospira platensis*	Hydrothermal carbonisation	190	36.7	Biofuel, nitrogen source (biofertiliser)	Yao et al. (2016)
*Gracilaria lemaneiformis*	Microwave-assisted low-temperature hydrothermal treatment,	160–200	16.3	Solid biofuel and levulinic acid	Cao et al. (2019)
*Chlorella vulgaris*	Fast pyrolysis	500	31	Biofertiliser	Wang et al. (2013a)
*Gracilaria edulis* *Eucheuma spinosum* *Kappaphycus alvarezii* *Saccharina japonica* *Sargassum* sp. *Undaria pinnatifida*	Slow pyrolysis	450	61.8, 61.7, 59.2, 49.7. 61.9, and 62.4%, respectively	Soil amelioration	Roberts et al. (2015)
*Chlorella vulgaris* ESP-31	Wet torrefaction	160–180	61.7–52.6	Biofuel	Bach et al. (2017)
*Chlorella vulgaris* FSP-E	Fast pyrolysis	500	26.9 ± 4.09	Improve soil fertility	Yu et al. (2018)
*Oedogonium intermedium*	Slow pyrolysis	450	29	Biofertiliser	Bach et al. (2017)
*Scenedesmus dimorphus*	Pyrolysis	300–600	36	Soil improvement	Bordoloi et al. (2016)
*Laminaria japonica*	Slow pyrolysis	200–800	78.34–27.95	Soil amendment, metal removal	Wang et al. (2013a)
Chlamydomans sp. JSC4	Torrefaction	200–300	93.9	Biofuel	Chen et al. (2016)
*Cladophora glomerate*	Fixed bed pyrolysis	400–600	44–31	Biofuel, biofertiliser	Norouzi et al. (2016)
*Fucus serratus*	Fluidised bed pyrolysis	500	29–36	Biofuel, biofertiliser	Yanik et al. (2013)
*Laminaria digitata*	Fluidised bed pyrolysis	500	29–36	Biofuel, biofertiliser	Yanik et al. (2013)

Biochar is produced through a variety of processes, including rapid, slow, and torrefaction, pyrolysis, hydrothermal, and microwave-assisted treatment. Regarding biochar yields, slow pyrolysis and torrefaction are more preferred (16.3–93.9%). The majority of biochar produced is used for soil amendment and biofuel refinery

Biochar produced has exceptional properties, including a large surface area, a high porosity, an aromatised carbon pattern, an abundance of functional groups, and a high mineral content. Biochar can be used in agronomy, animal farming, biogas production, water treatment, composting, construction, energy storage, soil remediation, and carbon sequestration due to biochar’s unique properties (Osman et al. 2022). Biochar derived from seaweed typically has a higher inorganic nutrient content, including calcium, phosphorus, magnesium, and potassium, than biochar derived from lignocellulosic biomass, which may be beneficial to soils and increase crop yield (Michalak et al. 2019; Sun et al. 2022).

In biochar applications, porosity and surface area are crucial parameters (Fawzy et al. 2021). Comparatively, seaweed biochar has lower surface areas than terrestrial-derived biochar, particularly woody feedstock. For instance, Michalak et al. (2019) and Roberts et al. (2015) stated that pyrolysis of *Eucheuma* and *Cladophora glomerata* produced biochars with specific surface areas of approximately 34.8 m^2^/g and 20 m^2^/g, respectively, while wheat straw-resulting biochar, rice husk-resulting biochar, and coconut shell-resulting biochar had comparatively high surface areas of about 256 m^2^/g (Medyńska-Juraszek et al. 2020), 280 m^2^/g (Tsai et al. 2021), and 152.8 m^2^/g (Zhao et al. 2019) respectively. However, the surface area of raw biochar derived from seaweed can be increased through additional pre-and/or post-treatment. For example, Zhou et al. (2018) found that potassium hydroxide pretreated kelp-derived biochar had a surface area of about 507.2 m^2^/g and porosity of 0.38 square centimetres/gram. These enhanced properties may provide additional advantages for enhancing the removal of contaminants, particularly in water treatment. Equally, water-washed *Ulva prolifera* biochar at 600 °C could improve seaweed biochar’s surface area from 13.46 m^2^/g for unwashed biochar to 257.41 m^2^/g (Yang et al. 2021b). Sun et al. (2022) described that lower ash content and higher pyrolysis temperature could increase the surface areas of seaweed’s biochar.

### Seaweed biochar as a carbon sequestration tool

Most volatile compounds in the feedstock are removed during the pyrolysis process; as a result, the resulting biochar is resistant to decomposition and highly stable (Farghali et al. 2022a; Bach and Chen 2017). Accordingly, biochar can be stored in soils for long periods, steadily resulting in the removal and sequestration of atmospheric carbon (Farghali et al. 2022a; Osman et al. 2022). Moreover, the high inorganic content of biochar may provide plant nutrients (Osman et al. 2022; Roberts et al. 2015; Fawzy et al. 2022). Additionally, the increased porosity of biochar may increase the soil's water-holding capacity, thereby enhancing the crop's water-use efficiency (Farghali et al. 2022a).

Biochar derived from seaweed has the potential to mitigate climate change by reducing greenhouse gas emissions. Some authors found that adding seaweed biochar to soils could boost the number of methane-oxidising microorganisms that reduce methane emissions from crop fields (Wu et al. 2019a; Wu et al. 2019b). Chubarenko et al. (2021) estimated that approximately 20–6000 tonnes of beach-cast seaweeds per kilometre of the shoreline might be collected annually in the southern Baltic Sea area. Consequently, the natural biodegradation of shoreline seaweed contributes significantly to greenhouse gas emissions. Thus, properly treating shoreline seaweed can reduce climate change and other problems such as eutrophication and strong odour (Lymperatou et al. 2022).

Wen et al. (2022) assessed the life cycle of beach-cast seaweed through pyrolysis. The authors found that pyrolysis of washed seaweed at 600 °C could result in carbon emission of—790.89 kg of carbon dioxides equivalent and negative overall energy demand of—2.98 gigajoules. In addition, at 600 °C stability of biochar over 100-year was 82% at 14.9 °C. Similarly, Sörbom (2020) reported that beach-cast seaweed-derived biochar had a significant capacity as a biofuel and carbon sequestration process. The author found that beach-cast has the ability to alleviate climate change by compensating 0.5 kg of carbon dioxide equivalent per kilogram of dry beach-cast, which was comparable to a carbon sequestration potential of 1600 tonnes carbon dioxide equivalent per year. In addition, this study demonstrated that forming biochar at an optimal temperature of 500 °C with optimised energy savings from natural drying decreased carbon dioxide equivalent emissions. Seaweed has a bio-charring conversion ratio of 48–57%, equivalent to high-quality plant biochar; thus, bio-charring can be a promising environment-friendly substitute to beach seaweed discarding by avoiding greenhouse gas emissions from biomass decomposition (Macreadie et al. 2017). As indicative of seaweed’s biochar carbon stability, Yang et al. (2021b) found that *Ulva prolifera* biochar had hydrogen/carbon and oxygen/carbon ratios of 1.025–0.173 and 0.480–0.193 compared to 1.956 and 0.897 for the raw seaweeds, respectively. Overall, the hydrogen-to-carbon ratio is the best indicator of biochar's environmental stability. For stabilised biochar, the upper limits of 0.4 and less than 0.7 for oxygen to carbon and hydrogen to carbon, respectively, are permitted, where biochar with an oxygen-to-carbon ratio of less than 0.2 is the most stable, with a half-life of greater than 1000 years; those with a ratio of 0.2–0.6 have a half-life of 100–1000 years; and those with a ratio of higher than 0.6 have a half-life of less than 100 years (Farghali et al. 2022a).

### Seaweed’s biochar for soil amendment

Seaweed biochar can also be used as a soil amendment in agronomy and forestry because the nutrients contained in seaweed are preserved and concentrated in biochar. Seaweeds are useful biofertilisers because they are rich in micronutrients, nitrogen, potassium, polysaccharides such as alginates, laminarin, carrageenans, and humic acid, and phytohormones (Yong et al. 2022; Nabti et al. 2016).

*Gracilariopsis funicularis* and *Laminaria* *pallida* seaweeds were pyrolysed at 200–800 °C. Pyrolysis at 400 °C temperature reduced biochar yields up to 50%, with even lower solid biochar yields at higher temperatures. *Gracilariopsis funicularis* seaweed biochar produced the highest macro-elements with a total carbon of 38.3%; nitrogen of 4.3%, and phosphorus of 6.3 g/kg, while *Laminaria pallida* biochar had the peak cations contents of 16.2 g/kg calcium; 6.4 g/kg magnesium; 151 g/kg potassium, and 45 g/kg sodium. The higher cadmium content of 3.9 milligrams per kilogram was problematic and exceeded the permitted biochar limits. Overall, a 400 °C pyrolysis temperature was optimum for the best quality biochar in aspects of total carbon pH, and macro-elements. *Gracilariopsi funicularis* biochar displayed substantially higher nutrient contents and thus has excellent potential in improving soil quality (Katakula et al. 2020). Roberts et al. (2015) concluded that seaweed-derived biochar had high nitrogen (0.3–2.8%), phosphorus (0.5–6.60 g/kg), and potassium (5.1–119 g/kg) contents and exchangeable cations. Therefore, using biochar derived from seaweed may reduce the demand for synthetic fertilisers, thereby reducing greenhouse gas emissions from fertiliser production. Table 11 provides an overview of the elemental composition of biochar derived from seaweed.

**Table 11:** Biochar production and elemental composition of seaweed

Species	Origin	Yield (%)	Carbon (%)	Hydrogen (%)	Oxygen (%)	Nitrogen (%)	Sulphur (%)	Heating value (megajoules/kilogram)	Phosphorus (gram/kilogram)	Potassium (gram/kilogram)	Carbon/nitrogen	References
*Gracilaria*	*South Sulawesi*	59.8	30.9	2.2	16.5	2.8	4.4	16.1	1.35	51.2	11	Roberts et al. (2015)
	*Java*	61.8	24.5	1.5	19.8	1.3	2.7	11.1	1.28	116	19	
*Eucheuma*	*South Sulawesi*	61.7	25.6	1.8	24.9	0.8	9.3	17.2	1.78	119	31	
	*Java*	57.2	23.7	1.2	20.6	0.7	7.0	14.6	0.91	163	33	
*Kappaphycus*	*South Sulawesi*	59.2	31.3	2.1	23.8	0.7	6.8	17.8	0.51	61.7	46	
	*Kiribati*	54.1	22.2	1.1	15.6	0.3	5.5	13.0	0.50	158	74	
*Saccharina*	*China*	49.7	28.0	1.9	16.4	2.2	1.0	11.4	4.69	8.9	13	
	*Korea*	45.3	35.0	2.4	18.4	2.4	1.6	14.8	6.60	51.9	15	
*Sargassum*	*China*	61.9	28.9	2.1	18.2	1.1	2.8	13.5	1.60	27.8	27	
	*Indonesia*	49.0	29.1	2.0	15.3	1.0	0.9	11.8	1.30	31.1	29	
*Undaria*	*China*	62.4	34.8	2.8	15.6	2.4	0.8	14.7	3.91	5.1	15	
	*Korea*	60.3	27.3	1.7	14.1	2.3	0.6	10.7	6.23	9.0	12	
*Saccharina japonica*	*Korea*	56.08	30.67	2.72	64.53	2.09	Not mentioned	2.73	Not mentioned	Not mentioned	14.7	Choi et al. (2017)
*Chlorella*	*Thailand*	45	10.00	3.6	28.1	0.9	0.9	4.8	Not mentioned	Not mentioned	11.11	Amin et al. (2020)
*Enteromorpha prolifer*	*China*	45.75	50.51	4.48	37.75	6.38	0.87	Not mentioned	Not mentioned	Not mentioned	7.92	Wang et al. (2020b)
Sargassum	Italy	41	34.86	5.20	27.50	4.25	1.07	14.19	Not mentioned	Not mentioned	Not mentioned	Taghavi et al. (2018)

The elemental composition of biochar derived from seaweed includes carbon, hydrogen, phosphorus, oxygen, nitrogen, nitrogen, sulphur, and potassium, among others. The variation in elemental composition can be attributed to the variety of seaweeds and the surrounding environment. The nitrogen, phosphorus, and potassium contents of seaweeds range from 0.7 to 6.38%, 0.51 to 6.60 g/kg, and 5.1 to 163 g/kg, respectively, making seaweed-derived biochar an excellent source of organic fertilisers that reduce the greenhouse gas emissions from synthetic fertilisers and a long-term carbon sink tool

Although seaweed biochar is regarded as a potential method for amending soil, seaweed-derived biochar’s practical application to soil is restricted by several factors. During pyrolysis, the low volatilisation temperature of sodium, sulphur, and chlorides, as well as the low melting temperature of sodium and potassium in seaweed, are obstacles that may lead to the formation of soil deposits and corrosion (Saber et al. 2016). In biochar, the non-volatile minerals remaining after pyrolysis would be preserved. The mineral content depends on the species and environment of the seaweed, where seaweeds from waters contaminated with heavy metals can have an adverse effect on crops and plants grown in different soil environments (Sun et al. 2022); for instance, higher content of iron, zinc, copper, manganese, cadmium, and mercury can produce toxic impacts on crop growth (Lee et al. 2020).

Furthermore, Roberts et al. (2015) found that seaweed-derived biochar typically has high levels of exchangeable sodium due to the aquatic growth of seaweed, which might cause soil salinity. To overcome these limitations, pre- and post-treatment processes have been suggested. For instance, Boakye et al. (2016) mentioned that seaweed pre-treatment through washing could decrease the toxicity level. Roberts et al. (2015) revealed that mixing seaweed-derived biochar with lignocellulosic-derived biochar could lower the sodium content and improve the carbon value of biochars mixtures, which lead to unique soil property fits the demands of the plant. Furthermore, Cole et al. (2017) indicated that composting a mixture of seaweed-derived biochar and sugarcane bagasse-derived biochar increased corn yields by 15%. The authors suggested that seaweed’s biochar might absorb unstable phosphorus and nitrogen to avoid nutrient losses in the soil and diminish the sodium content. However, the current pre- and post-treatment techniques may increase the final outcome's cost. Optimising the pyrolysis conditions for maximum mineral retention capacities and bioavailability of heavy metals in biochar is, therefore, urgent and requires additional research.

## Summary

Several thermochemical methods, such as pyrolysis, hydrothermal carbonisation, and torrefaction, can be used to produce seaweed biochar from seaweed biomass. Biochar is an efficient method for sequestering carbon and improving soil quality. Applying biochar to the soil can also improve soil quality by increasing the soil's water-holding capacity, nutrient-holding capacity, and microbial population. Zhu et al. (2017) proposed direct and indirect reasons for the improvement of soils following the addition of algal biochar. The direct causes are associated with the physicochemical properties of biochar, such as biochar’s structure, surface area, and porosity, which provide shelter for soil microbiota, as well as the nutrients retained in biochar that are essential for the growth of soil microbes. The potential of biochar to reduce the toxicity of volatile organic compounds and stable free radicals is a further direct cause. The indirect effects of algal biochar on soils can be attributed to the biochar's capacity to alter soil pH, provide aeration, stimulate enzymatic activity, influence soil elemental cycling, and reduce soil contaminants, thereby protecting soil microbiota from toxicants.

## Influence of seaweed extracts on crop health and production

In addition to climate change, extensive use of chemical pesticides has accelerated the occurrence of resistant, infectious pathogens and pests affecting important crops, resulting in substantial losses in crop production (Yong et al. 2022). Seaweed extract can be used to enhance crop productivity. For instance, Ali et al. (2021) demonstrated that seaweed extract could increase plant productivity, overcome pest resistance, abiotic stresses, including salinity and drought, and substantially change plant and soil microbiome, hence supporting sustainable plant growth. In addition, seaweed-supplemented soil significantly improves crop health and productivity by enhancing root and shoot elongation, enhancing nutrient and water uptake rate, boosting seed germination, and conveying plant resistance to frost, salinity, and phytopathogenic agents, such as bacteria, parasites, insects, fungi, and other pests (Yong et al. 2022; Nabti et al. 2017; Williams et al. 2021).

Seaweeds are effective biofertilisers because they are rich in nitrogen, potassium, humic acid, micronutrients, polysaccharides such as alginates, laminarin, and carrageenans, and other growth-promoting phytohormones (Nabti et al. 2016; du Jardin 2015). Specifically, treatment of tomato plants and sweet pepper with *Ascophyllum nodosum* extracts combined with safe fungicides produced the highest total plant yield (57% increase) and the lowest disease levels (60% reduction) compared to their individual application, indicating the beneficial and synergistic effects of seaweed extracts on the plant (Ali et al. 2021). Seaweed induction of disease suppression was attributed to stimulation of peroxidase, phenylalanine ammonia-lyase, polyphenol oxidase, chitinase, total phenolic, β-1,3-glucanase, and higher *PinII* and *ETR-1* genes expressions (Ali et al. 2021). Similarly, seaweed extracts used in foliage and soil significantly affected the phyllosphere and rhizosphere microbial components and improved cross-microbial-linkage that considerably impacts plant health and production (Ali et al. 2021). Experimentally, using seaweed fertilisers such as *Ascophyllum nodosum* extracts in the rhizosphere soil of pepper, maize, and tomato crops altered microbial communities and diversity structures on the plant roots and soil (Wang et al. 2018a; Chen et al. 2020). Nevertheless, seaweed’s chemical ingredients vary among seasons and also among different environmental stimuli, including salinity, nutrient availability, temperature, and light, which present challenges in developing effective biofertilisers (Yong et al. 2022). Further concerns regarding the availability and accessibility of seaweed biofertilisers for manufacturers and farmers, such as the versatility, benefit/cost analysis, and compatibility with agricultural machinery and practices, especially compared with chemical fertilisers, are recommended (Yong et al. 2022).

## Seaweed-derived bioplastic

Due to their low production costs and substantial characteristics, such as low density, corrosion resistance, and durability, plastic products are indispensable in modern life. The global production of plastics reached 322 gigatonnes in 2015, which has steadily increased over time (Lopez et al. 2018). Conventional plastics are non-biodegradable; therefore, the continuous disposal of plastic in landfills poses significant risks to organisms and contributes to environmental issues such as greenhouse gas emissions, water pollution, explosion risk, and hygienic issues. The Center for International Environmental Law estimated that 850 megatonnes of greenhouse gas emissions were caused by the production and combustion of plastics. By 2050, annual plastics emissions could reach 2.75 gigatonnes of carbon dioxide equivalent (Center-for-International-Environmental-Law 2019). To date, plastic management has been a significant obstacle that must be overcome to reduce greenhouse gas emissions and climate change.

Due to their biodegradability by soil microorganisms and reduced adverse environmental impacts, bioplastics offer advanced sustainable alternatives, making them a potential solution to the global plastic waste problem (Folino et al. 2020; Emadian et al. 2017; Butbunchu and Pathom-Aree 2019). In addition, bioplastics are renewable sources derived from animals, plants, and microorganisms, and they exhibit several advantages over conventional plastics, such as lower energy requirements, less reliance on fossil fuels, and fewer pollutants released during the biodegradation process (Qasim et al. 2021). Bioplastics derived from renewable sources include agropolymers, bacterial polymers, and algal polymers (Devadas et al. 2021; Dang et al. 2022). On the other hand, plant-based bioplastics, such as maize and corn, are promising but require land repurposing for plastics production rather than food production (Yong et al. 2022). Some species of bacteria accumulate intracellular polyhydroxyalkanoate particles as carbon and energy resources within their cells, making them another source for bioplastic production. Despite this, their applications are limited due to cultivation difficulties and low biomass yields (Chia et al. 2020). Thus, bioplastics account for approximately 1% of the 368 gigatonnes of plastics produced annually (Dang et al. 2022).

Bioplastics derived from seaweed could be a promising area of plastics production research over agro-polymers biomass. Where seaweed biomass contains less lignin and more long-chain hydrocarbons than terrestrial plants (21–31% lignin, 26–43% cellulose, and 30% hemicelluloses), high-purity cellulose can be extracted economically to make bioplastics (Dang et al. 2022; Zanchetta et al. 2021; Yang et al. 2019). In addition, seaweeds are distinguished by a rapid growth rate, a diverse cultivation environment, and the absence of a requirement for arable land (Yong et al. 2022; Dang et al. 2022).

**Fig. 6:**
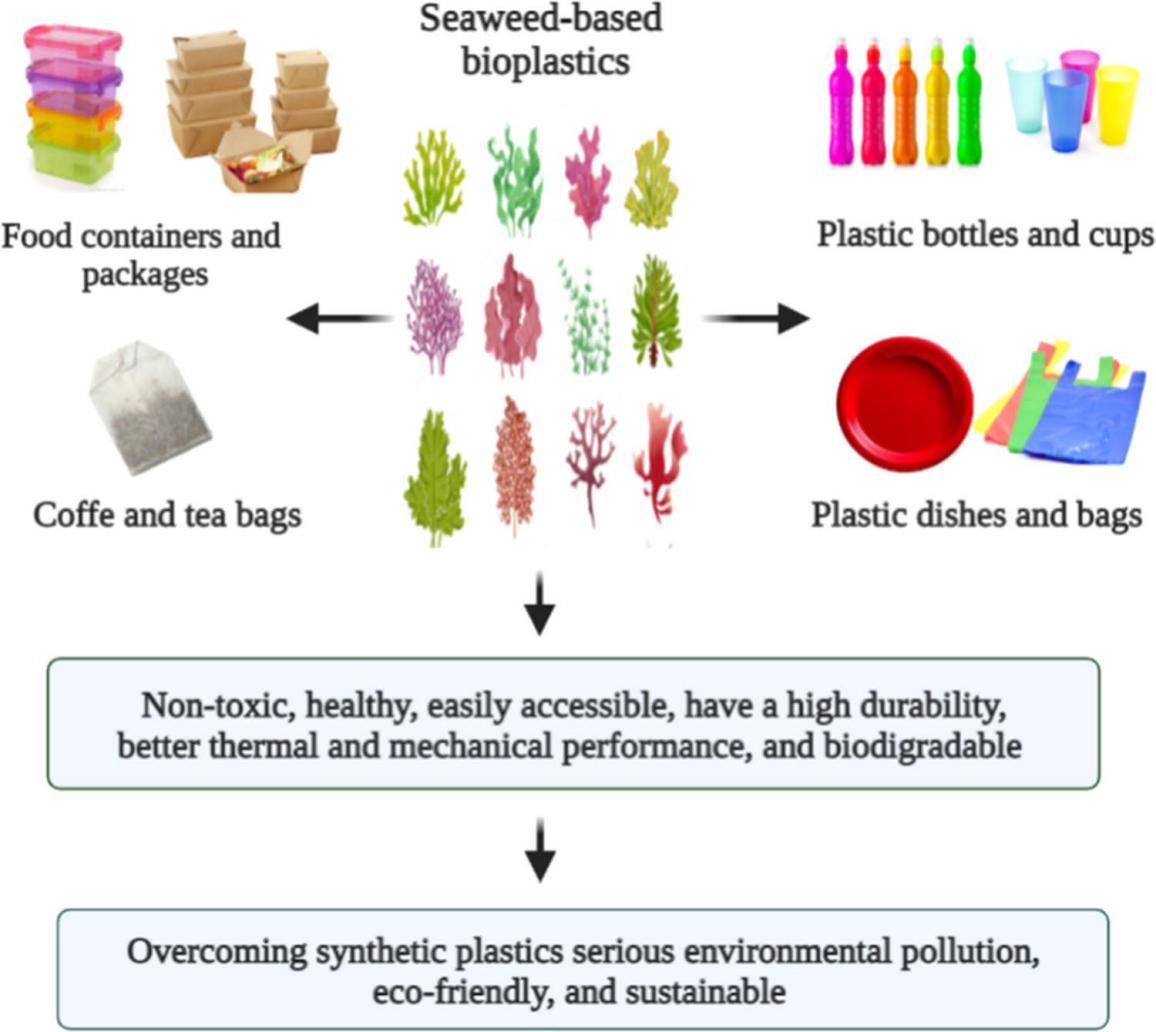
Bioplastic products made from seaweed. In the food industry, seaweed polysaccharides can be purified and used to make a variety of bioplastic products. The resulting bioplastics are safe, non-toxic, and have superior durability and mechanical performance. In addition, bioplastics are biodegradable and recyclable, providing the environment with sustainable, green, and eco-friendly plastics

Seaweed-derived polysaccharides, such as agar, alginate, and carrageenan, may be recognised as bioplastic precursors and utilised in bioplastics manufacturing (Tavassoli-Kafrani et al. 2016). Therefore, polysaccharides bioplastics derived from seaweeds are promising polymers due to their biocompatibility, safety, high durability, and superior thermal and mechanical performance (Joye and McClements 2014; Lomartire et al. 2022; Mouritsen et al. 2021). As shown in Fig. 6, seaweed-derived bioactive extracts are ideal candidates for sustainable packaging production. Seaweed polysaccharides, such as carrageenans and alginates, could be used as biopolymeric films and biodegradable packaging materials with positive health benefits, thereby overcoming severe environmental pollution that negatively affects microplastics and severe environmental pollution that negatively affects ecosystems (Lomartire et al. 2022), where seaweed-based bioplastics are more environmentally friendly than petroleum-based plastics (Folino et al. 2020).

Several seaweeds have been used for bioplastic biofilm productions due to their higher polysaccharide contents, including red seaweeds (*Eucheuma*, *Kappaphycus*, *Gracilaria*, *Porphyra*, *Pterocladia*, and *Gelidium*), green seaweeds (*Enteromorpha*, *Ulva*, and *Codium*), and brown seaweeds (*Laminaria*, *Lessonia*, *Macrocystis*, and *Ascophyllum*) (Lomartire et al. 2022; Freile-Pelegrín and Madera-Santana 2017). Lim et al. (2018) mixed the alginate compound from *Sargassum siliquosum* with sorbitol, sago starch, and calcium chloride to synthesise bioplastic films. Their findings indicated that the films produced using mixtures of two grams of alginate powder from *seaweeds* and 15% of sorbitol treated with 75% calcium chloride possessed sufficient bioplastic film characteristics. Doh et al. (2020) used cellulose nanocrystals from *Sargassum natans* and *Laminaria japonica* to prepare bioplastic films. The authors found that the presence of cellulose nanocrystals improved the film's physicochemical, thermal, and mechanical characteristics, thereby providing suitable green bio-packaging. Other authors produced bioplastics from *Kappaphycus* seaweed (Sudhakar et al. 2021), *Eucheuma cottonii* (Wullandari et al. 2021), *Ulva lactuca* (Guidara et al. 2019), and others packing bioplastics (Lomartire et al. 2022).

### Summary

The use of seaweed and seaweed extracts in synthesising biodegradable bioplastics is gaining popularity. This natural bioplastic could reduce global plastic pollution by more than 300 gigatonnes per year. Additionally, seaweed-derived components' antimicrobial and antioxidant properties will extend the shelf life of water, medications, and foods. Several industries have shown interest in using seaweed for safe, recyclable, and hygienic packaging, not only for healthy foods and supplements but also for eco-friendly and sustainable packaging that preserves food and maintains food characteristics. However, additional research is required to reduce bioplastics' operation and extraction costs for sustainable and extensive uses.

## Seaweed as a functional ingredient for the food industry

**Fig. 7:**
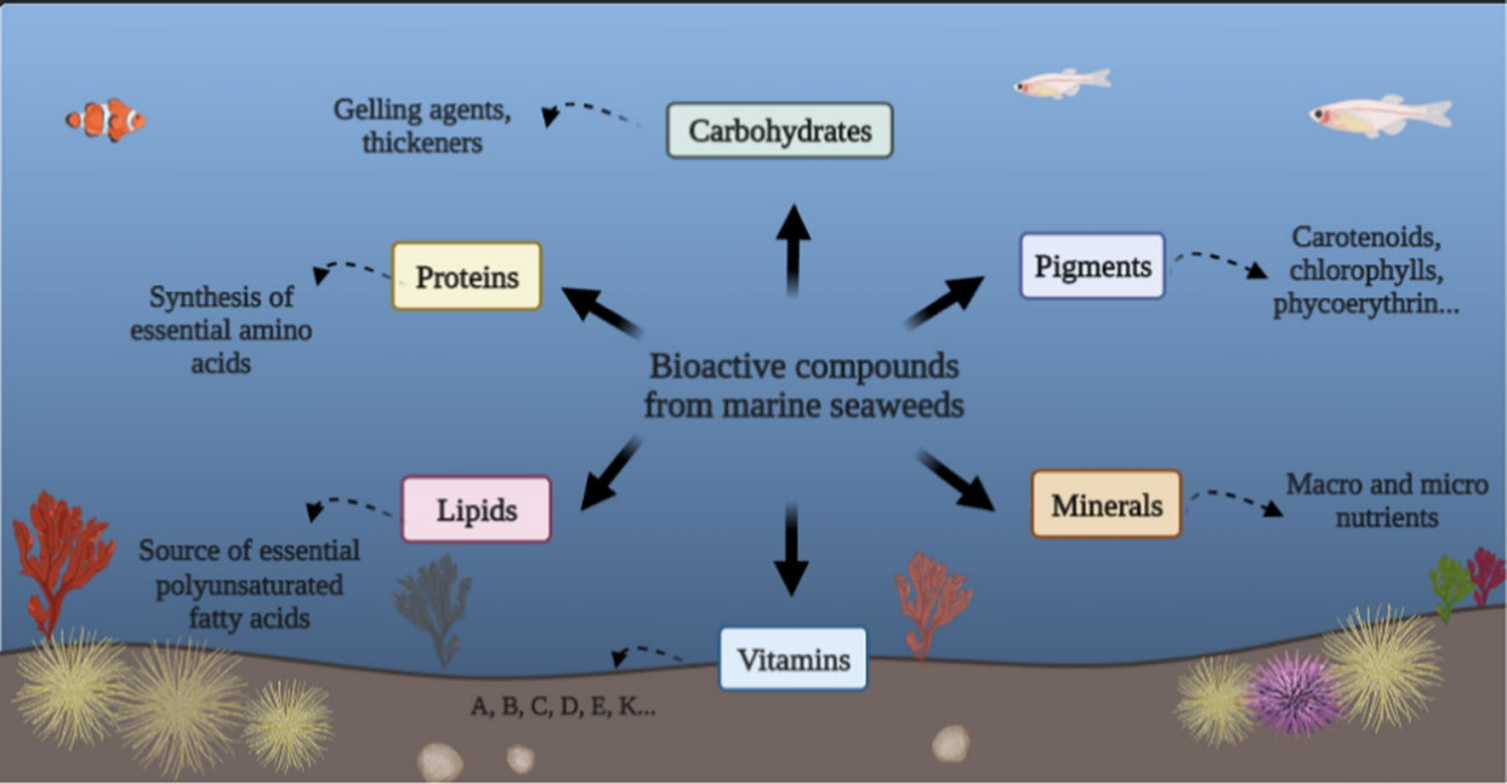
Phytonutrients and bioactive compounds found in marine algae. Approximately 40% of the biomass of seaweed is comprised of carbohydrates. Protein content in seaweeds varies by geographic region, species, season, and growth conditions. In brown, green, and red seaweeds, protein concentrations range from 4 to 10%, 15*–*25%, and 8*–*40%, respectively. Lipid is of great interest because lipid contains essential fatty acids, such as omega 3. Comparatively, fewer lipids are present in seaweed than in other terrestrial plants. Typically, the lipid content of brown seaweed ranges between 1 and 4.5 grams per 100 grams of dry seaweed. The amount of algal ash varies between 8.7 and 66.07% of the dry matter. Minerals such as potassium, sodium, magnesium, phosphorus, and calcium are abundant in seaweeds, as are trace elements such as copper, iron, zinc, chlorine, iodine, and manganese. Pigments, such as water-soluble phycobiliproteins harvested from red seaweed, fucoxanthin, a xanthophyll pigment in brown seaweed, and chlorophyll-a are also found in seaweeds

The dietary food value was early quoted 460 Before Christ by Hippocrates, who stated the common words of “*Let food be thy medicine and medicine be thy food.*” Consumer demand for healthy, nutrient-dense foods with multiple functions has increased steadily in recent years (Granato et al. 2020). However, industrialisation has been associated with a new lifestyle characterised by fast-food consumption and sedentarism, which increase the prevalence of diseases such as obesity and cardiovascular disorders. Government policy and the food industry must be more decisive and concerned in order to overcome the current lifestyle attitude. Recent interest has been focussed on food product reformulation through the elimination, reduction, and substitution of certain food components with other, healthier constituents, among the various strategies for addressing this public health issue (Heck et al. 2017; Cofrades et al. 2017). As shown in Table 3 and Fig. 7, seaweeds are relatively low in fats and lipids compared to other healthy food additives. Seaweeds also contain a significant amount of proteins, carbohydrates, dietary fibres, bioactive components, and minerals. The nutritional value of seaweeds is significantly influenced by seasonal variation and geographic location (Schmid et al. 2018), water salinity and temperature (Nielsen et al. 2016), farming techniques (Sharma et al. 2018), and other factors.

### Seaweeds applications in food products

The food industry is advancing rapidly with multibillion-dollar budgets as manufacturers, entrepreneurs, food companies, and scientists strive to deliver the best food, food by-products, and food components to optimise this lucrative sector. Seaweed has been utilised in food production for many years, thriving through the finding and utilising hydrocolloids as food additives. Today, seaweed hydrocolloids and edible seaweed production are seaweed’s most prominent raw materials applications. In 2015, 35 nations accounted for 80% of the global seaweed trade, importing approximately 251,709 metric tonnes of dried seaweeds valued at 634 billion United States dollars (FAO 2018). In addition, the global sales of agar, carrageenan, and alginates were estimated at 93,035 metric tonnes, an increase of about 3%, and 1.58 billion United States dollars (Porse and Rudolph 2017). Hydrocolloids derived from seaweed are frequently utilised in food industries such as bakery, confectionery, milk, and meat products.

On the other hand, incorporating beneficial seaweed ingredients such as minerals, dietary fibres, fucosterol, fatty acids, and fucoxanthin directly into existing foods through the use of semi-processed seaweeds or seaweed powders is deemed to be the most effective method. The incorporation of seaweed ingredients or whole seaweeds as useful fractions improves the nutritional and textural qualities of food products such as bakery, meat, and dairy products and provides health benefits against certain chronic diseases, such as diabetes, hypertension, obesity, and dyslipidemia (Roohinejad et al. 2017; Sharma and Baskaran 2021). Bread is an indispensable food item consumed globally. Adding seaweed extract and powder to bread modifies the dough characteristics (stickiness and water absorption) and increases the bread's dietary fibre content; other beneficial ingredients, such as renin and alginate, act as satiety inducers and cardioprotective substances (Porse and Rudolph 2017; Fitzgerald et al. 2014). The incorporation of brown and red seaweed as food ingredients has been reported to range from 0.5 to 8% in bread, 5–20% in pasta, 3–30% in noodles, 2.5–20% in cake, 5–60% in biscuits, 3–9% in cookies, and 3.5% in extruded maize (Quitral et al. 2022). Additionally, the presence of biologically active fucoxanthin (0.02–0.23 mg/g) and fucosterol (0.51–2.55 mg/g) had antioxidant, anti-diabetic, and anti-cancer effects on the products (Wang et al. 2018b; Gutierrez-Rodriguez et al. 2018; Lee et al. 2021). In addition, adding seaweed ingredients to meat products improves the properties of meat products and makes meat healthier, which is advantageous for many meat consumers (Gullón et al. 2021). Specifically, incorporating seaweed’s polyphenols into meat products increases meat's antioxidant properties and shelf life by delaying lipid peroxidation in muscle tissues and increasing protein in diets without altering an individual's eating habits. (Wang et al. 2022). Functional seaweed ingredients, including alginate and fucoidan, retain their properties when added to beverages and milk. Alginate extracted from *Laminaria hyperborean–*brown seaweed, for instance, may induce insulinemia, hypoglycemia, and appetite suppression in healthy adults (Torres et al. 2020). Thus, future use of alginates derived from seaweed could potentially manage type-2 diabetes and obesity.

### Seaweeds as prebiotics and gut health promotion

Several disorders, including autoimmune and allergic diseases, inflammatory bowel disease, diabetes, and obesity, can be caused by dysbiosis, a gut microbial imbalance syndrome (Cristofori et al. 2021). Contrarily, healthy microbiota aids nutrient absorption and protects against dysbiosis-related diseases and other metabolic disorders (Sommer et al. 2017). Therefore, the use of dietary additives, such as prebiotics, in the improvement of gut microbes to promote health is well established and is currently a component of holistic and comprehensive efforts to manage diseases and enhance welfare (Salminen et al. 2021; Houghton et al. 2018; Krumbeck et al. 2016). Among various probiotic substances, the insoluble and soluble dietary fibres, including oligosaccharides originating from seaweeds, have demonstrated a positive effect on the intestinal microbiota. For example, the hydrolysis of agars and alginate from brown seaweed *Enteromorpha prolifera* and *Ascophyllum nodosum* and red seaweed *Gelidium sesquipidale* and *Glacilaria* sp., produced polysaccharides of low molecular weight that induce prebiotic activity in vitro which in turn balanced human gut flora by improving positive microbes such as *Lactobacillus* and *Bifidobacterium* (Kong et al. 2016). The obtained polysaccharides also increased total short-chain fatty acids, a valuable bacterial molecule that influences multiple human physiological processes, including gluconeogenesis, neurogenesis, inflammation, and central nervous system functions (Kong et al. 2016).

Besides, certain polysaccharides derived from brown seaweed, such as glucan- and alginate-based laminarin, modulated the microbiota in pigs and rats (Ho Do et al. 2021). In addition to regulating microbial functional metabolites, seaweed polysaccharides may help reduce uric acid, triglycerides and increase the antioxidant profile in serum and short chain fatty acid production in animal models' faeces (Cañedo-Castro et al. 2019). The authors added that the indigestible polysaccharides in seaweeds may pass through the small intestine and end up in the large intestine, where they may be degraded by intestinal microbiota. Therefore, seaweed-derived polysaccharides play an innovative role in promoting gut health, making them a promising functional prebiotic that could be incorporated into human and animal foods. To clarify the exact chemistry and mechanism of polysaccharides, as well as to apply bioinformatics analysis to untargeted metabolites that could interpret additional flora pathways and biomarkers acting to promote and regulate host health and manage disorders, additional research is required.

## Uses of seaweed in livestock feed

Animal protein is an essential nutrient source for livestock. Thus, sustaining high nutrient and protein sources is urgently required for sustainable livestock production. Globally, 1 billion tonnes of feed are used in livestock production, resulting in a $400 billion profit in 2016 (IFIF 2020). Due to the wide distribution of biomass and seaweed's high fat and protein content relative to legumes and grains, seaweeds have long been utilised as animal feed in Europe. More biorefining and processing is suggested to increase the conversion of seaweeds into useful protein mass and other animal feed ingredients (Makkar et al. 2016). For example, Bikker et al. (2016) clarified that using *Ulva latuca* green seaweed increased the protein contents from 225 g/kg (dry matter basis) to 343 g/kg through the use of enzymatic hydrolysis with hot water treatments as biorefinery approaches. Likewise, the biorefinery of *Ulva ohnoi* increased the protein content of dry seaweed from 22.2 to 39.5–45.5% (Magnusson et al. 2019). The direct addition of seaweed biomass into animal feed can also improve the livestock's health and growth and the subsequent meat quality. Supplementing *Undaria pinnatifida* into swine feed improved the total immune response via regulating the toll-like receptor genes and cytokines, in addition to modulating the intestinal microflora to promote the beneficial *Lactobacillus* bacteria and reduce *Escherichia coli* consortium (Shimazu et al. 2019). The effects of feeding seaweeds to various animal species will be discussed in depth.

### Ruminants

The utilisation of seaweeds in ruminant rations is a promising feeding strategy due to the high ruminant demand for protein in feed, the demand for substituting conventional soybean and other animal proteins, and the need to comply with animal feeding-related food market regulations. The majority of research on the potential use of seaweeds in ruminants has focussed on the addition of trace amounts of various seaweed species to animal diets and the subsequent evaluation of animal performance, health status, and product quality. Specifically, feeding sheep with 1–5% *Ascophyllum nodosum* brown seaweed in the daily ration could balance the ruminal microbiota and decrease *Escherichia coli* population (Zhou et al. 2017). Furthermore, feeds supplemented with *Ulva lactuca* of approximately 20% of feed did not alter the animal palatability. In addition, this supplementation reduced the protein degradability to 40% and moderated energy digestibility to 60%, which is similar to low- to medium-quality forages and is appropriate to use with rations of high energy or low protein content as cereal grains (Morais et al. 2020). Meanwhile, feeding *Ulva*, *Ruppia*, or *Chaetomorpha* could be incorporated into sheep diets up to 30% on a dry matter basis without showing any adverse impacts on growth performance or feed digestibility (Rjiba-Ktita et al. 2019).

Red seaweed has gained more interest, particularly in ruminant feed (Mahrose and Michalak 2022). For example, using 70% concentrated *Phymatolithon calcareum* extract at a rate of 0.5 g/kg feed, buffered the rumen pH but neither enhanced fibre digestion nor altered rumen fermentation (Morais et al. 2020). This could be attributed to the high ash content of seaweeds; therefore, using seaweed with a lower ash content would make seaweed an excellent choice for future research.

Supplementation of *Ascophyllum nodosum* brown seaweed to cattle feed could reduce faecal shedding of *Escherichia coli* (Makkar et al. 2016). Some brown seaweeds are fed to ruminants, especially sheep. For instance, *Laminaria hyperborean*, *Laminaria digitata*, and *Saccharina latissima* are accountable for 90% of the sheep’s summer ration, which meets approximately 13% of crude protein. Some authors found that feeding *Macrocystis pyrifera* up to 30% of goat did not affect degradability, digestibility, rumen fermentation parameters like ammoniac nitrogen and pH, and increased urine excretion and water intake (Makkar et al. 2016). Therefore, it could be concluded that ruminant diets could contain up to 30% seaweed without compromising feed intake, digestibility, or growth performance (Morais et al. 2020).

### Poultry

Typically, poultry are raised for two primary functions: meat production (broiler poultry) and egg production (laying poultry). The addition of seaweed to poultry production will therefore be addressed in two distinct ways.

Feed for broilers has primarily consisted of soybean and corn meals, with corn being the predominant energy source due to corn’s digestibility and availability (60–75% of feed). Historically, high corn prices necessitated the development of novel feed ingredients to provide broilers with the necessary nutrients to maintain productivity and reduce feed prices (Morais et al. 2020). Due to seaweed’s high macro/micro-element content, seaweed has the potential to be a unique poultry feed, thereby enhancing the performance and growth of poultry as well as the egg and meat quality (Michalak and Mahrose 2020; Cardoso et al. 2019). In addition, the probiotic bioactivities of seaweed polysaccharides may improve poultry performance, health, and egg quality. In addition, seaweeds may improve chicken meat and increase the omega-3 fatty acid content of eggs (Abu Hafsa et al. 2019). Adding *Sargassum muticum* at 5–15% ratios to the broiler diets steadily improved the body weight, feed conversion ratio, and average daily gain (Erum et al. 2017). The authors concluded that supplementing poultry diets with a greater proportion of seaweed increased the average daily gain.

Furthermore, Bai et al. (2019) revealed that supplementing 1% *Laminaria japonica* powder to broiler diets improved the feed conversion rate owing to the improved dietary energy value. The utilisation of *Ulva rigida* at ratios of 2–6% as prebiotics to improve bird's growth performance (Cañedo-Castro et al. 2019). The authors observed no significant differences in the body weights of the birds; however, there were significant differences in feed consumption, feed conversion ratio, and mortality rates. Due to the properties of seaweed, feed consumption rates increased in broilers fed 4 and 6% *Ulva rigida*; however, mortality rates were higher in the chicken groups fed 6% seaweeds compared to the control groups (Cañedo-Castro et al. 2019).

Michalak and Mahrose (2020) stated that feeding broilers with diets containing 1–2% *Sargassum wightii* received the highest evaluation scores from consumers regarding colour, flavour, juiciness, tenderness, and taste. Moreover, adding *Sargassum wightii* at ratios of 1–4% to broiler diets enhanced carcass traits of broilers, such as leg weight, thigh, breast, and dressing. The optimum additive effects were observed for *Sargassum wightii* dosed at 1 and 2%, which were attributed to the specific properties of seaweed, including vitamins, minerals, essential amino acids, sterols, polyunsaturated fatty acids, and polysaccharides. Furthermore, the inclusion of broiler diets with 0.3% of *Chondrus crispus* red seaweed powder markedly enhanced the breast and carcass yields and reduced the abdominal fat contents (Martínez et al. 2019). However, seasonal variations in seaweed's nutritional composition must be considered.

Eggs contain proteins, vitamins, minerals, and lipids of high quality. Age, genetics, and diet can affect the nutritional value and composition of eggs. Therefore, egg enhancement can be achieved by adding seaweed to the poultry diet, which increases levels of vitamins, minerals, and fatty acids (Choi et al. 2018; Baniamerian et al. 2019). For example, the inclusion of *Ulva* green seaweed in poultry diets at ratios of 1–3% improved egg quality and yield, increased egg weight, yolk colour, shell thickness, decreased yolk cholesterol, and reduced feed conversion ratio. In addition, seaweed extracts reduced faecal *Escherichia coli* counts, suggesting improved health benefits (Morais et al. 2020; Wang et al. 2013b). Moreover, *Chondrus crispus* red seaweed has been applied at 2–4%/feed to decrease *Salmonella enteritidis* level, an infectious bacterium which spreads vertically from layers to eggs via the ovarian-oviducts pathway or through contaminated faeces (Morais et al. 2020). Using *Sargassum* sp., brown seaweed at a 3–6% feed could decrease yolk cholesterol and triglycerides, enhance egg quality, lutein plus zeaxanthin, and carotene contents (Michalak and Mahrose 2020). However, these outcomes need more studies to determine the bioavailability and best concentration of seaweed extracts.

### Fish farming

Fish feeds are estimated to account for 50% of operating costs in an intensive fish farming system (Additives et al. 2019). Therefore, finding inexpensive alternatives for aquaculture feed, particularly terrestrial plants such as oilseed crops and legumes, is necessary. Seaweed can be used as a nutritionally and economically advantageous substitute for soybeans in fish meals, given that soybeans do not completely satisfy the nutritional needs of fish (Chirapart and Ruangchuay 2022). Recent research has demonstrated that seaweeds are a promising nutritional option for fish aquaculture. Morais et al. (2020) used red *Gracilaria* and green *Ulva* seaweeds to lower nutrient contents in seawater effluents pollution and expand feed sources aiming at changing market resources as an extra-source income. They concluded that using seaweeds in aquaculture sectors can advance the aquaculture industry and reduce the dangers of oligotrophic seas with a high level of biodiversity. Integrating fish or shrimp aquaculture with seaweeds, sea urchins, and/or bivalves to retain extra nutrients from shrimp/fish discharge tanks is a recently implemented technology. Through this integration, seaweeds can purify the nutrient-rich effluents discharged from fish/shrimp cages or tanks, thereby creating a new source of aquatic feed. In addition, this integration is a tool for ecosystem balance that prevents environmental pollution from aquaculture effluents and increases the seaweed’s value (Hasselstrom et al. 2018). Diverse seaweeds are used as aquaculture feed sources and metabolites in the integration systems, including *Ulva* sp., *Gracilaria* sp., *Ascophyllum nodosum, Laminaria digitata,* and *Sargassum sp*. (Thepot et al. 2021). Lomartire et al. (2021) stated that using seaweeds as fish diet supplements enhanced the physiological activity, growth, lipid metabolism, carcass quality, disease-fighting, and stress response of several fish species.

Moreover, using *Saccharina latissimi* as a feed additive could ameliorate fish farming and enhance fish resistance against oxidative stress (Kamunde et al. 2019). The authors also explored the opportunity of using *Laminaria* sp. seaweed as a salmon meal. They found that salmon fed with seaweed showed improved intake, plasma antioxidant capacity, growth performance, and mitochondrial respiration; in addition to feeding, seaweeds could alleviate atmospheric temperature increases (Kamunde et al. 2019). They added that decreasing crude protein and minerals after replacing salmon’s diet with 10% seaweeds had no adverse impacts on salmon smolts. Consequently, adding brown seaweeds to aquafeeds could present a cost-effective and optimum approach favouring the aquaculture industry (Kamunde et al. 2019). In conclusion, using seaweed to increase the yield of fish farms is possible. The advantages may include increased growth rate, monetary gain, disease resistance, and ecological conservation. More research is required to optimise the use of seaweeds as aquafeeds by experimenting with various types and combinations of seaweeds.

### Summary

Seaweeds have unique characteristics and chemical structures that allow them to be utilised in a variety of contexts. The high protein content of seaweeds, for instance, can be utilised as animal, fish, and poultry feed to combat the escalating feed cost. This may not only aid in reducing feed costs but also improve the quality of meat, milk, and egg products. On the other hand, using seaweed as animal feed for up to 30% can improve the health and immune status of livestock against a variety of diseases. Seaweeds can be utilised as poultry feeds to improve the immune status of broilers, increase meat production, and decrease the microbial load in the digestive tract. Using different seaweed species (brown, red, or green) can improve poultry egg qualities, including weight, quality, and cholesterol reduction in the yolk; other biomolecules extracted from seaweeds can reduce toxic bacterial levels in the poultry.

Interestingly, using brown, red, and green seaweed mixtures to improve eggs would be a promising supplement that requires further study. Additionally, seaweeds can be integrated with fish farms to clean the nutrient-rich effluents discharged from aquaculture cages or to provide a new source of aquatic feeds that improve the health status, weight, and meat quality of fish. As a result, this type of integration provides a tool for balancing ecosystems, which could prevent environmental pollution from aquaculture effluents.

## Seaweed’s use in pharmaceuticals and cosmetics

**Fig. 8:**
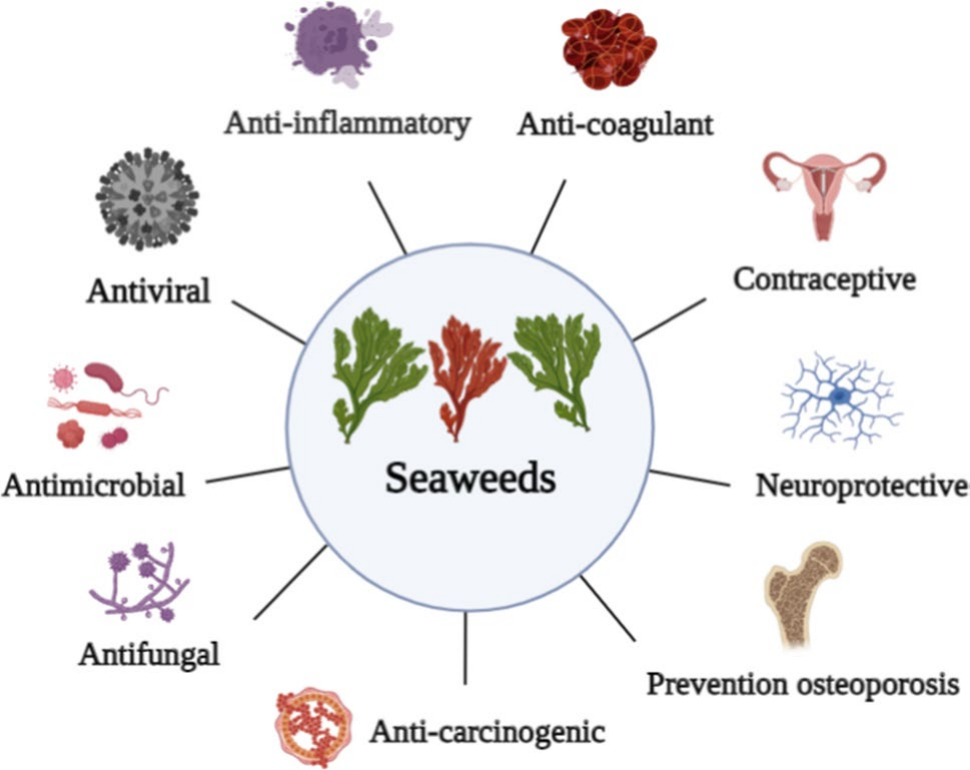
Biological activities of seaweed. Antimicrobial, antifungal, antioxidant, anticoagulant, anti-inflammatory, and mosquitocidal agents are among the biological processes in which seaweed extracts can be utilised. Secondary metabolites derived from seaweed, such as polysaccharide fucoidan, sulfoquinovosildiacyl-glycerols, and caulerpin, exhibit a broad range of biological activities

Several secondary bioactive metabolites with considerable therapeutic and industrial potential can be extracted from seaweeds (Table 3 and Fig. 8). The bioactivities of seaweed metabolites include antifungal, antimicrobial, antiviral, contraceptive, anti-inflammatory, anticancer, antioxidant, and anticoagulant properties (Gomez-Zavaglia et al. 2019; Khan et al. 2022). To optimise the beneficial use of seaweed metabolite activities for human health, extraction efficiency in separating and enhancing the required bioactive components is required, along with the best cultivation practices. In addition, the suitability of the extracted biomolecule for industrial and pharmaceutical applications should be legitimised through an appropriate clinical evaluation that includes a safety outline. Recent advances in metagenomics, genomics, proteomics, molecular biology, and bioinformatics assays may contribute significantly to discovering new pharmaceuticals from seaweeds.

### Antibacterial, antifungal, and antagonistic properties of seaweed

Antimicrobials are one of the most important medical interventions required worldwide to treat diseases. However, the occurrence of antimicrobial resistance against numerous pathogens has a negative impact on this therapeutic success and may endanger the patient's life (Tarin-Pello et al. 2022; Razzaque 2021). Therefore, searching for novel antimicrobials without resistance is of critical clinical importance. Several secondary biomolecules found in seaweeds, such as polysaccharide fucoidan, sulfoquinovosildiacyl-glycerols, and caulerpin, exhibit extensive biological activity, including antimicrobial activity (Table 12). Antimicrobial compounds derived from seaweeds are predominantly components of the seaweed's natural defence mechanism against invading pathogens (Bhowmick et al. 2020; Polat et al. 2021). For example, several compounds, including phenols, volatile halogenated hydrocarbons, terpenes, indoles, acetogenins, and fatty acids, have been extracted from seaweeds assembled from the coastlines of Egypt and showed antibacterial activity against multi-drug resistant microbes such as *Pseudomonas aeruginosa*, *Staphylococcus aureus*, *Escherichia coli, Shigella flexneri*, *Corynebacterium* sp., and *Klebsiella pneumoniae* (El Shafay et al. 2016). In addition, seaweed extracts have shown antibacterial effects against some pathogens, such as vancomycin-resistant *Enterococcus faecalis* and methicillin-resistant *Staphylococcus aureus* (Asharaf et al. 2022). This intervention could be a hopeful finding not only to fight against pathogens but also to diminish antibiotic usage in the poultry sector, where antimicrobial resistance frequently occurs. Extracts of *Ceramium rubrum, Cladophora vagabunda,* and *Ulva rigida* have been applied to suppress *Candida albican*, *Bacillus cereus*, and *Escherichia coli* growth in the aquatic environment (Sirakov et al. 2019).

**Table 12:** Characteristics and functions of seaweeds and their bioactive compounds

Seaweed type	Component/extract	Technical function/property	References
*Pterocladiella*, *Gelidium amansii,* *Pterocladia*, *Gracilaria*	Agar	Antioxidant, thickener	Casas et al. (2016), Xu et al. (2018), Zhang et al. (2019b), Zhang et al. (2019c)
Brown seaweeds	Alginate	Thickening agent, high stability, gelling agent	Morais et al. (2021)
Red seaweeds, *Gracilaria chouae*, *Porphyra haitanensis*, *Gracilaria blodgettii*	Carrageenans	Anticancer, antioxidant, antiaging, radiation protection, thickeners properties	Priyan Shanura Fernando et al. (2019a, b), Sun et al. (2015), Fernando et al. (2019a, b), Xie et al. (2020)
Fucoidan (Sigma), *Ascophyllum nodosum*, *Ecklonia maxima*, *Saccharina japonica*, *Hizikia fusiforme*, *Sargassum hemiphyllum*, *Chnoospora minima*, *Sargassum polycystum*, *Sargassum horneri*, *Sargassum vachellianum*	Fucoidans	Photo-ageing inhibition, anti-inflammatory: elastase and collagenase inhibition, skin-whitening: minimised elastase activity, antioxidant	Jesumani et al. (2019a), Pangestuti et al. (2021), Jesumani et al. (2019b), Shanura Fernando et al. (2018), Ruocco et al. (2016), Sanjeewa et al. (2018), Wang et al. (2021b), Wang et al. (2020c), Su et al. (2020)
*Saccharina japonica*, *Codium* *tomentosum*, *Chondrus crispus*	Polysaccharides	Hydration	Wang et al. (2015)
*Ulva* sp.	Ulvan	Antiaging	Jiang et al. (2020), Fourniere et al. (2021)
*Agarophyton chilense*, *Champia novae-zelandiae*, and *Pyropia plicata*	Eleven mycosporine-like amino acids	Ultraviolet protection, antioxidant	Orfanoudaki et al. (2019)
*Iridaea cordata*, *Curdieara covitzae*	Mycosporine-like amino acids extract (palythine and asterina-330)	Ultraviolet protection, antioxidant, anti-ageing	Rangel et al. (2020)
*Gracilaria vermiculophylla*	Mycosporine-like amino acids extract (shinorine, asterina-330 palythine, and porphyra-334)	Ultraviolet protection, antioxidant	Barceló-Villalobos et al. (2017)
*Rhodymenia pseudopalmata*	Mycosporine- amino acids extract (deoxygadusol, palythene, and usujirene)	Antioxidant	Pliego-Cortés et al. (2019)
*Porphyra umbilicalis*	Extracts-rich in proteins, minerals, vitamins, shinorine, and porphyra-334	Skin protective, hydration, anti-roughness, anti-wrinkle	Gianeti and Maia Campos (2014)
*Ecklonia cava*	Dioxinodehydroeckol	Prevent ultraviolet-induced apoptosis	Ryu et al. (2015)
*Ecklonia stolonifera*, *Eisenia bicyclis*	Eckol	Anti-inflammatory	Manandhar et al. (2019a), Manandhar et al. (2019b), Eom et al. (2017)
*Chondrus crispus*, *Palmaria palmata*, *Mastocarpus stellatus*	Mycosporine-like amino acids extract (mainly asterina-330, palythine, shinorine, palythinol, usujirene, and porphyra-334)	Antioxidant	Athukorala et al. (2016)
*Pyropia yezoensis*	Peptide	Anti-inflammatory	Lee et al. (2015)
*Porphyra haitanesis*	Hydrolysed extract	Anticancer	Fan et al. (2017)
*Ecklonia stolonifera*, *Ecklonia cava*	Eckol, 8,8′-bieckol, 6,6′-bieckol, phlorofucofuroeckol-A, and dieckol	Antiallergic	Sugiura et al. (2021)
*Ecklonia stolonifera, Okamura*	Fucofuroeckol-A	ultraviolet protection	Vo et al. (2018)
*Cystoseira compressa*	Fuhalol	Antioxidant	Gheda et al. (2021)
*Fucus vesiculosus*	Fucophloroethol (isomer)	Antioxidant	Hermund et al. (2018)
*Ishige foliacea*	Octaphlorethol A	Antioxidant	Lee et al. (2014)
Eisenia bicyclis	Phlorofucofuroeckol A	Antioxidant and hepatoprotective effect	Kim et al. (2011)
Brown algae, *Fucus serratus*, *Ascophyllum nodosum*, *Halidrys siliquosa*, *Himanthalia elongata*	Phlorotannins	Anticoagulant, photoprotective, antioxidant, anti-inflammatory, antiviral, anticancer, antibacterial, antidiabetic	Casas et al. (2016), Gheda et al. (2021)
*Hizikia fusiformis*	Fucosterol	Anti-photodamage: ultraviolet protective, Anti-inflammatory	Sun et al. (2015), Hwang et al. (2014)
Commercial	Phytosterol	Anticancer	Zhangfan et al. (2019)
*Ulva rigida*, *Gracilaria*, *Saccharina latissima*, *Fucus vesiculosus*	Lipid profile	Antioxidant	Neto et al. (2018)
*Pterocladia capillacea*, *Ulva* *lactuca*, *Sargassum hornschuchii*	Fatty acid profiling	Bioindicator of chemical stress	Mohy El-Din (2018b)

Antimicrobial, anticancer, antioxidant, anti-inflammatory, anti-ageing, skin-whitening, gelling and thickening agents, ultraviolet protection, and other medical applications are possible with seaweed metabolites. The most frequently employed extracts are agar, alginate, carrageenans, fucoidans, phlorotannins, and ulvan. The biomolecules derived from nature can serve as a substitute for chemically synthesised agents that maintain an individual's health and reduce the environmental risks associated with chemical synthesis

Besides controlling bacterial infection, managing pathogenic fungal growth and infection is likewise important to agricultural and clinical areas, where pathogenic fungal infections have a vital adverse effect on crops and produce massive economic losses; meanwhile, increase the susceptibility of human beings to the occurrence of hazardous fungal toxins that are harmful at minute dosages (Moretti et al. 2017). Specifically, Padam and Chye (2020) stated that seaweed extracts from *Caulerpa racemose*, *Gracilaria edulis*, and *Sargassum myriocystum* had antagonistic impacts on plant fungus, where seaweed’s extracts produce inhibition and retardation of *Alternaria porri* mycelial growth on onion crops. Similarly, ulvan extracted from *Ulva fasciata* disrupted the development of *Stemphylium solani* fungus (Reis et al. 2018), while the extracted polyunsaturated fatty acid ethyl esters (ethyl nonadecadienoate and ethyl tetracosapentaenoate) from *Laurencia okamurai* could suppress some pathogenic fungi infecting human, including *Aspergillus fumigatus, Trichophyton rubrum, Candida glabrata,* and *Cryptococcus neoformans* (Padam and Chye 2020).

### Antiviral and mosquitocidal activities of seaweed

Seaweed and seaweed extracts have antiviral properties against a variety of viruses (Lomartire and Goncalves 2022). Seaweed metabolites can function as antiviral agents by boosting the host's immune system or inhibiting virus replication prior to virus entry into host cells (Lomartire and Goncalves 2022). Seaweeds can target several viruses, such as herpes viruses, lentivirus, influenza viruses, and coronaviruses, and others (Lomartire and Goncalves 2022; Wei et al. 2022). The antiviral activity of polyphenols and sulphated polysaccharides was greater than that of other seaweed compounds (Table 12). The virucidal effect of sulphated polysaccharides is based on their interference with initial viral attachment to the negatively charged host cell surface. Negatively charged sulphated polysaccharides can interact with positively charged viral glycoproteins, preventing the virus from entering the target cell (Lomartire and Goncalves 2022). For instance, sulphated polysaccharides such as galactan, carrageenan, fucoidan, ulvan, alginate, naviculan, and calcium spirulan extracted from seaweeds are found to produce inhibitory activities against cell damages generated by several viruses (Wei et al. 2022). Pagarete et al. (2021) exploited the antiviral potential of seaweeds over 50 years of technological and scientific developments in the field of seaweed antivirals. The authors conducted a survey of 16 clinical trials, a bibliometric investigation of 999 systematic references, and an analysis of 84 patents and observed that seaweeds have a diverse range of biomolecules that displayed marked antiviral effects, including carrageenan. The authors concluded that antiviral applications of seaweed extract have been successfully commercialised and have significant expansion potential. Thus, natural polysaccharides derived from seaweed could be a promising antiviral agent and a safe alternative to synthetic ones (Jabeen et al. 2021).

Several vector-borne diseases could also be treated with ingredients derived from seaweed. Dengue viral disease transmitted by mosquitoes is one of the most common diseases that cause epidemics and kill many people yearly, particularly in India. *Aedes albopictus* and *Aedes aegypti* sp. are the most frequent carriers of the dengue virus. In addition, *Aedes* sp. can transmit other related viruses like chikungunya and zika (Freile-Pelegrín and Tasdemir 2019). The proliferation of the chikungunya virus begins with virus attachment to the host cell's surface. Consequently, inhibiting virus-host attachment and binding could be a helpful approach to managing the virus. Seaweed polysaccharides have the ability to modify cell surface characteristics; hence, using seaweed-derived polysaccharides represents an effective method to avert some deadly viral infections. For example, Rodrigues et al. (2017) extracted *ulvan*n from *Caulerpa cupressoidesis* seaweed that contained 11% sulphate and 6% uronic acids. The seaweed-ulvan extract has shown antiviral activity against dengue virus type 1 in a cell line study with a reasonable selective index (more than 714) and no cell cytotoxicity.

Of special concern, mosquitocidal activities of seaweed-originated compounds have been demonstrated against mosquitoes, the vector of almost viruses. Yu et al. (2014) stated that halogenated sesquiterpene-elatol-derived from *Laurencia dendroidea* red seaweed exhibited effective larvicidal influences against *Aedes aegypti* with a mortality rate of more than 91% at 50 parts per million. The authors added that *Cladophora glomerata*-derived fatty acids, including myristic, palmitoleic, lauric, and capric acids, were found to have 3–14 ppm lethal concentration_50_ against *Aedes triseriatus*. Similarly, Salvador-Neto et al. (2016) reported that *Laurencia dendroidea* derived elatol and halogenated sesquiterpene, (+)-obtusol caused larvicidal mortality of 30% and 9%, respectively, at 10 parts per million concentrations against *A. aegypti* within 24 h. Additionally, obtusol extracts have shown concentration-dependent larvicidal activities. To this end, seaweed extract has promising antiviral and mosquitocidal activities, and more research is needed against more agents with different integrations and applied protocols.

### Antioxidant activity of seaweed

In highly oxidative conditions, seaweed has demonstrated potent antioxidant systems. As a photosynthetic organism, seaweed is exposed to high levels of oxygen and light, which allow the formation of free radicals and other powerful oxidising components (Penalver et al. 2020). However, seaweed develops robust defences against oxidative agents, as evidenced by the absence of oxidative damage to the chloroplast's thylakoid membranes (Penalver et al. 2020), where 301 macroalgal metabolites are known to have antioxidant activities (Tziveleka et al. 2021), such as sulphated polysaccharides, polyphenols, unsaturated fats, amino acids, and peptides, which present various antioxidant traits (Table 12). Additionally, Tziveleka et al. (2021) classified the antioxidant metabolites present in algae into phenolic molecules, comprising phlorotannins, bromophenols, and flavonoids; nitrogenous compounds, comprising peptides; terpenoids, comprising steroids and carotenoids; chlorophyll-derived pigments and alkaloids; as well as carbohydrates and polysaccharides. Among various antioxidants, the most significant are secondary metabolites in seaweeds, fucoidans, phlorotannins, and carotenoids (Hermund 2018). Phlorotannins may be used as an effective replacement synthetic antioxidant in the food refinery (Hermund 2018). The bioavailability of hydroxyl groups in phlorotannins structure and phloroglucinol units oligomerisation are accountable for phlorotannins' antioxidant action (Hermund 2018). Furthermore, seaweeds comprise polyphenols with particular bioactivity, which may influence gene expression (Hoseinifar et al. 2022). Thus, there is much scientific interest in using seaweed to prevent ageing, cancer, and cardiovascular disease (Penalver et al. 2020; Tziveleka et al. 2021).

Brown seaweed polysaccharides, such as alginic acid, laminarans, and fucoidans, exhibited potent antioxidant activity (Afonso et al. 2019), hence considered powerful antioxidant agents. The antioxidant properties of sulphated polysaccharides depend on various aspects such as molecular weight, sulphation degree, sugar type, and glycosidic bonds (Koutsaviti et al. 2018). Specifically, seaweeds containing low molecular polysaccharides pose more antioxidant ability than high molecular weight containing types (Liu and Sun 2020) explained that the low molecular weight polysaccharides could be integrated more easily into the cells and provide protons more effectively than the high molecular weight polysaccharides (Penalver et al. 2020). Likewise, a positive relation was shown between sulphates and antioxidant activity in the fucoidan portion of *Saccharina japonica* brown algae (Ajisaka et al. 2016; Kalasariya et al. 2021). Sargachromanol E obtained from *Sargassum horneri* showed scavenging capability against reactive oxidant species and protected cells from oxidative damage in ultraviolet-exposed humanoid fibroblasts (Jesumani et al. 2019a). Carotenoids are also reported to be other effective antioxidants in seaweeds (Hermund 2018), with Xanthophyll and tocopherols the most abundant carotenoids. Xanthophylls are effective quenchers of singlet oxygen, while tocopherols are extensively utilised in the food industry owing to their effective free radical scavenging ability.

### Immunomodulatory and anti-inflammatory properties of seaweed

The immune system and inflammatory response are the body's natural protective mechanisms for managing injuries, combating infections, restoring homeostasis, and healing wounds (Sattler 2017). However, prolonged and unwarranted inflammation induced by infectious agents and necrotic cells must be controlled to prevent detrimental tissue effects (Broggi and Granucci 2015). In addition to essential oils, the anti-inflammatory properties of seaweeds have been attributed to polyunsaturated fatty acids, sulphated polysaccharides, fucoxanthin, alkaloid, and astaxanthin (Padam and Chye 2020; Rajauria et al. 2017; Kang et al. 2016). Tabarsa et al. (2018) found that water-soluble sulphated polysaccharides obtained from a green seaweed, *Ulva intestinalis*, could improve the immune-modulatory activity on RAW264.7 macrophage cells, generating a high quantity of nitric oxide and proinflammatory cytokines such as tumour necrosis factor-α, interleukin-1β, interleukin-12, and interleukin-6. The authors showed that the existence of anti-inflammatory cytokines via the expression of the interleukin-10 gene prevented severe inflammatory effects. Equally, sulphated fucan obtained from *Agarum cribrosum* released considerable interleukin-10, cyclooxygenase-2, and nitric oxide that stimulated RAW264.7 macrophage cells and interleukin-10.

Additionally, fucoidan derived from *Sargassum fusiforme* could disrupt the P-selectin function, which is an important protein for the binding of leukocytes to the endothelium during an acute inflammatory response (Wu et al. 2019c). *Kappaphycus alverazii* was displayed anti-inflammatory activity compared to commercial antihistaminic medicine “Loratadine” in controlling asthma symptoms in rats, reducing mucus generation and downregulations of proinflammation genes (Anyanji et al. 2015). This can provide a promising option to the useful benefits of seaweed for reducing chronic asthmatic patients’ symptoms.

### Anticancer properties of seaweed

Cancer is one of the leading causes of death worldwide. In addition, the cost of cancer treatment in the European Union has increased dramatically from 35.7 billion euros in 1995 to 83.2 billion euros in 2014 (Jonsson et al. 2016). Chemotherapy for cancer has negative effects on neighbouring normal cells; therefore, efforts to discover effective, novel, and non-toxic chemotherapeutics from natural resources are crucial, especially in aquatic environments (Vaikundamoorthy et al. 2018). Due to the presence of certain components, such as carotenoid fucoxanthin and sulphated polysaccharides, as shown in Table 12, seaweeds could be used as anticancer agents. Several studies found a strong correlation between the phenolic antioxidant capacity of seaweeds and their anticancer properties (Lee et al. 2021; Kalasariya et al. 2021; Ferdous and Balia Yusof 2021; Sakthivel and Devi 2019). Vaikundamoorthy et al. (2018) reported that polysaccharides extracted from brown seaweed, *Sargassum wightii*, exhibited a substantial decline in the propagation of human mammary carcinoma cell lines (Michigan Cancer Foundation-7 and M.D. Anderson—Metastatic Breast 231) in a dose-dependent manner.

Similarly, sulphated laminarans obtained from the brown seaweeds (*Fucus evanescens*, *Saccharina japonica,* and *Saccharina cichorioides*) have shown a wide range of anticancer activities that suppressed the breast adenocarcinoma migration through the inhibition of the metalloproteinases 9 and 2 matrixes activities (Malyarenko et al. 2016). In addition to polysaccharides, polyphenolic phloroglucinol and fucoxanthin extracted from seaweeds were capable of suppressing two human colorectal cancer cell lines (human colorectal carcinoma-116 and human colon adenocarcinoma cell-29) due to deoxyribonucleic acid damages in cancer cells and presented no hostile impacts on a normal colon cell line-18Co (Lopes-Costa et al. 2017). A synergistic effect was found when both seaweeds derivatives were combined with the antimetabolite drug 5-fluorouracil, increasing the commercial drug potency. Fucoxanthin is frequently present in several kinds of brown seaweeds that display not only anticancer characteristics but also anti-inflammatory and antioxidant activity (Rajauria et al. 2017). Alarif et al. (2016) isolated three sesquiterpenoids chabrolidione B, eudesma-4(15),7-diene-5,11-diol, and teuhetenone from *Laurencia obtuse* red seaweed that displayed antiproliferative activity against Michigan Cancer Foundation-7 cell lines, with teuhetenone being the most favourable component. Despite some promising findings, most anticancer research on seaweeds is still in the infancy stage, and more studies are required to be conducted on humans.

### Seaweed as anticoagulant material

In the biomedical industry, heparin is the most widely used anticoagulant medication for the treatment of thromboembolic disorders. Thrombocytopenia, a haemorrhagic effect associated with heparin, is nonetheless the most commonly reported adverse effect. This necessitates the search for additional antithrombotic agents (Padam and Chye 2020). Some reports suggested that seaweed polysaccharides could exhibit anticoagulant activity, in addition to being free of any dangerous viruses or prions that are known to contaminate commercial heparins (Faggio et al. 2016). Polysaccharides extracted from seaweed are also safe for cellular metabolism, highlighting seaweed extracts more than commercial heparin. Pharmacologically, the pathway of seaweed polysaccharides is entirely dependent on the sulphate group’s presence, position, and molecular weight. Fucoidans, phlorotannins, and sulphated polysaccharides derived from brown algae have been identified in the literature as anticoagulant agents (Liu et al. 2018).

Furthermore, *ulvans* from green algae and carrageenans from red algae also have anticoagulant properties in vitro models. Sulphated polysaccharides from *Agardhiella subulata* and *Ulva fasciata* prolonged the coagulation time activities on human blood after using prothrombin time and partial thromboplastin time assay (Faggio et al. 2016). However, sulphated polysaccharides' efficacy and safety profile should be evaluated comprehensively and broadly in vivo.

### Seaweed as contraceptive material

Several types of seaweed exhibited diverse contraceptive properties. For example, red algae collected from coastal waters in Sri Lanka, namely *Gracilaria corticata* and *Gelidiella acerosa* exhibited potent post-copulatory contraceptive capability in female rats without displaying any adverse effects (Dolui 2021). Similarly, some authors have reported the potential contraceptive activity of ethanol-extracted *Gracilaria edulis* and *Gracilaria corticata* seaweeds in the mouse model (Aziz et al. 2020; Martins et al. 2014). In addition, *Gracilaria edulis* ethanolic extracts showed a 100% inhibition of sperm motility*,* presenting a spermicidal agent that disrupts sperm plasma membranes (Dolui 2021). Likewise, *Halimeda gracilis* demonstrated a 100% suppression of human sperms due to sperm’s plasma membrane being damaged at a dosage of 10 mg/ml after 20 s of contact time. A phytochemical assessment of *Halimeda gracilis* extracts revealed the existence of secondary metabolites, including sugar, flavonoids, alkaloids, and protein (Prakash et al. 2014).

### Use of seaweed in cosmetics

Modern westernised lifestyle attitudes and fashions are accelerating the expansion of the global cosmetics market. Due to the inefficacy of synthetics and a shift in attitude towards products derived from natural sources, the cosmetics industry has recently incorporated natural bioactive ingredients (Jesumani et al. 2019a). Several studies cautioned against the use of cosmetics derived from synthetic materials, highlighted their toxicity concerning an increase in adverse effects following their application, and deemed them dangerous health products for consumers (Kalasariya et al. 2021). Urgently recommended is the search for and testing of cosmetics derived from natural ingredients to meet the needs of consumers and protect their health (Thiyagarasaiyar et al. 2020). Seaweeds can be used as natural cosmetics or incorporated into cosmetic formulas due to their novel chemical structure and constituents (Ariede et al. 2017). However, natural cosmetics derived from seaweed may also adversely affect humans (Thiyagarasaiyar et al. 2020). Even so, natural-derived ingredients are less hazardous than synthetic ingredients and have greater bioactivities with minimal cytotoxicity effects in humans (Thiyagarasaiyar et al. 2020; Alvarez-Gomez et al. 2019). Specifically, seaweeds can be used in the cosmetics industry as bioactive constituents, texturing stabilisers or emulsifiers, organic dyes, and materials of skincare-relevant biomolecules (Pimentel et al. 2017). Since seaweeds are photosynthetic organisms, they frequently produce secondary metabolites that protect the seaweed's cells and organelles from ultraviolet rays, allowing them to be used as photoprotective ingredients in sunblock. Table 12 demonstrates that the extracted seaweed ingredients have a high potential for incorporation into cosmetic formulations and improvement of the cosmetic industry (Lopez-Hortas et al. 2021). In addition, several studies have described the multiple positive effects of seaweed compounds as topical products with low cytotoxicity on the skin (Alvarez-Gomez et al. 2019; Wang et al. 2015; Kageyama and Waditee-Sirisattha 2019).

Seaweeds can be incorporated into skin-whitening, anti-ageing, and anti-pigmentation formulations (Polat et al. 2021; Thiyagarasaiyar et al. 2020). In particular, *Fucus*, *Laminaria*, and *Chondrus* seaweeds are primarily used to nourish and rehydrate the skin (Jesumani et al. 2019a). Pangestuti et al. (2021) reported that seaweed bioactive components such as laminarin, fucoidan, mycosporine carrageenan, amino acids, and fucoxanthin have unique functional properties and are commonly used as cosmetics products and anti-photoaging properties. These bioactive compounds showed anti-photoaging characteristics mediated by intracellular reactive oxygen species scavenging bioactivity in ultraviolet irradiated cells and in vivo experiments (Pangestuti et al. 2021). Cellulite is also a cosmetic problem and may be relieved by routine skincare to enhance the visual look of the skin. Laminaran polysaccharide obtained from *Laminaria* is mainly used as an anticellulite product due to laminaran’s wide range of bioactive assets (Morais et al. 2020). Fucoidan has the potential to be applied as an anti-ageing agent because fucoidan can improve the elasticity and hydration of cells by promoting the synthesis of heparin-growth factor, which improves the growth of tissues and cells (Dolui 2021; Pangestuti et al. 2021).

As they provide protection against sunburn, sun-induced pigmentation, and tanned skin, skin-whitening and sunscreen products are advancing rapidly. Tyrosinase catalyses the conversion of l-tyrosine to 3, 4-dihydroxy-l-phenylalanine, which is then oxidised to dopaquinone and converted to melanin. Exposure to ultraviolet light increases the production of both melanosomes and tyrosinase. Ingredients derived from seaweed can inhibit tyrosinase and are generally recommended for skin whitening (Lopez-Hortas et al. 2021; Manandhar et al. 2019a; Manandhar et al. 2019b). Brown seaweed extracts are effective as kojic acid, commonly used as a skin-lightening ingredient (Arguelles and Sapin 2020; Arguelles 2021). Likewise, Park et al. (2021) indicated that *Pyropia yezoensis* extracts could be used as effective and safe materials to improve skin whitening and prevent skin wrinkle formation, where the extracts exhibited marked reduction in tyrosinase activity, enhanced collagen synthesis, and promoted skin brightness in a study of 23 volunteers.

Alginic acid is a polysaccharide that is present in numerous brown seaweeds. Alginic acid inhibits scar formation and promotes wound healing; consequently, alginate is combined with collagen in the clinical industry to repair tissues (Kuznetsova et al. 2020). Alginate can be converted from insoluble to soluble forms by combining it with sodium or potassium salts, particularly at a low pH, allowing alginates to be utilised in hydrogel formation (Kuznetsova et al. 2020). Consequently, alginate is widely used in variable gelling agents in cosmeceuticals and pharmaceuticals, such as protective colloids, emulsion stabilisers, ointment bases, lotion, pomades, hand jellies, hair products, facial cream, and beauty masks (Dolui 2021; Lopez-Hortas et al. 2021; Kuznetsova et al. 2020).

Carrageenan is another polysaccharide that is found in several red seaweeds such as *Chondrus crispus, Betaphycus gelatinum, Kappaphycus alvarezii, Eucheuma denticulatum, Hypnea musciformis, Gigartina skottsbergii, Mastocarpus stellatus, Sarcothalia crispata,* and *Mazzaella laminaroides*. Generally, there are three chief carrageenan kinds, including iota (ι), lambda (λ), and kappa (κ). Kappa and iota carrageenans present gelling traits, while lambda carrageenan is used as a viscosifier/thickening agent (Kalasariya et al. 2021). Carrageenans are found in several personal grooming and hygiene by-products such as toothpaste, hair conditioners, medicines, lotions, deodorants, shampoos, foams, shaving creams, sunray filters, and sprays. About 20% of carrageenan products are used in pharmacy and cosmetology (Morais et al. 2021). However, it is still difficult to design a new formula with seaweed ingredients due to the incompatibility of the ingredients with the cosmetic formulation in terms of colour, odour, and consistency. Incorporating seaweed into cosmetic formulations still requires additional research and development to produce the most effective cosmetic products.

### Summary

It is possible to obtain and utilise seaweed extracts in numerous subsidiary products, including antimicrobial, antifungal, antiviral, and anti-inflammatory products. This can reduce reliance on chemical products and their resistance problems, such as antimicrobial resistance, thereby improving human and animal health. In addition, seaweed extracts exhibit antiviral activities against a broad spectrum of viruses, including coronavirus, either by direct virucidal effect and blocking virus attachment receptors or by enhancing the host's immune system.

Extensive exposure of human skin to environmental stressors, such as solar radiation, pollutants, and chemical cosmeceutical ingredients, increases the production of reactive oxygen species, leading to various skin-damaging issues, including carcinogenesis, ageing, wrinkles, dark circles, dullness, and age spots. Bio-purified components derived from seaweed have proven to be highly beneficial in cosmetic formulations, where seaweed-based products can serve as natural substitutes for synthetic compounds. Primary and secondary seaweed metabolites generated as a natural defence against invaders can be used as bioactive ingredients in cosmetics, such as antiaging, anti-acne, deodorising, antimicrobials, antioxidant, moisturising, whitening agent, anti-wrinkle, anti-inflammatory, sensory enhancer, ultraviolet protection, anti-allergic, stabiliser, viscosifier, and thickeners. The natural utilisation of marine seaweeds and biomolecules derived from seaweed is essential for humankind. However, monitoring the biochemical properties of seaweed-based extracts remains an issue that must be resolved. Thus, advancing seaweed cultivation methods and establishing environmentally friendly extraction techniques could yield promising research results. Additionally, collaborative research with numerous national and international cosmetic companies can promote analytical methods of seaweed screening for safety, efficacy, and long-term suitability, thereby enhancing consumer safety and confidence in marine seaweed-based bioactive cosmetic products.

## Conclusion

This review critically investigates the potential use of seaweed in various biorefineries. Seaweeds have the potential to be used as a carbon sink in aquatic oceans or as substrates for anaerobic digestion and biochar production if they are utilised as such. By transferring to the deep ocean or becoming buried in sediments, seaweed carbons can be released and sink. Thus, seaweeds provide a solution to climate change. On the other hand, sequestering carbon from seaweed as biomethane through anaerobic digestion can achieve the carbon sequestration concept that verifies the principles of climate change mitigation. In addition, biomethane can be utilised as a bioenergy source to replace fossil fuels, thereby resolving the current energy crisis. Seaweed biomass or seaweeds converted to biochar can serve as carbon sinks and biofertilisers that replace synthetic fertilisers, thereby mitigating climate change. Seaweeds can also remove pollutants from wastewater, increase the pH of water, provide oxygen to the ocean's waters to reduce ocean acidification and deoxygenation, and absorb nutrients and pollutants from water bodies. Bioplastics derived from seaweed are an emerging technology to combat plastics pollution and replace synthetic plastics.

Seaweed and seaweed extracts may be promising human foods or additives for promoting gut microbiota as prebiotic agents and enhancing resistance/fighting against various microorganisms, including coronavirus. In addition, seaweeds can be promising livestock protein sources to replace the reliance on other costly protein sources, thereby reducing the price of meat, milk, and other animal products and optimising their cost. Additionally, seaweed and seaweed extracts can improve the quality of meat, eggs, and milk, providing additional benefits. As a natural alternative to soybeans for fish meals, seaweed can be incorporated into fish/shrimp farms for nutritional and economic benefits.

Due to their unique properties, seaweeds can be utilised in various other contexts. For example, seaweed bioactive compounds can be extracted and utilised in a variety of products, such as antimicrobial, antioxidant, anticancer, antifungal, antiviral, anti-inflammatory, and antioxidant products. Using seaweed in medical products could reduce reliance on chemical products and their resistance issues, such as antimicrobial resistance, thereby improving the health of humans and livestock. The overexposure of human skin to solar radiation, pollutants, and cosmeceutical ingredients derived from chemicals increases the production of reactive oxygen species, resulting in various skin problems. As anti-ageing, anti-acne, deodorising, moisturising, whitening agent, anti-wrinkle, anti-inflammatory, sensory enhancer, ultraviolet protection, anti-allergic, stabiliser, viscosifying, and thickening agents, purified metabolites derived from seaweeds could be used as natural components in synthetic cosmetics and medicines.

To manage market demand and overcome obstacles such as planning constraints, policy constraints, financial constraints, and market constraints, seaweed manufacturers require the support of policymakers. Industrial limitations include a lack of awareness of seaweed applications in a variety of fields and the absence of a comprehensive policy regarding the use of seaweeds at the national and international levels. Seaweeds must be utilised globally, and more knowledge must be gained from Asian countries that dominate seaweed cultivation and production. Applications of seaweeds as a climate change mitigation strategy can swiftly decide on seaweeds' extensive cultivation. Future research must identify seaweeds as alternative foods and bioactive chemical components in order to increase seaweed production and the value of seaweed products. Researchers must also highlight the limitations of bioactive compound extractions and seek cost-effective ways to solubilise valuable biomolecules and use bioactive compounds as natural ingredients in various fields. Carbon-sequestration-based life cycle assessment of seaweeds from cultivation to consumption is also required to evaluate the balance of carbon sequestration by seaweed habitats and total carbon release over the lifecycle of seaweed production.
